# Supplementary material for: Emergence of fractal geometries in the evolution of a metabolic enzyme
Source: Nature. 2024 Apr 10;628(8009):894–900. doi: 10.1038/s41586-024-07287-2 (PMC11041685; doi:10.1038/s41586-024-07287-2)
Supplement: Supplementary file 1 — This file contains Supplementary Figs. 1–6 and Tables 1–3. [file 41586_2024_7287_MOESM1_ESM.pdf]

nature portfolio

https://doi.org/10.1038/s41586-024-07287-2

Supplementary information

Emergence of fractal geometries in the evolution of a metabolic enzyme

In the format provided by the authors and unedited

This file contains:

Supplementary Figure 1 | Uncropped SDS-PAGE Gel images and uncropped plates from survival assays

Supplementary Figure 2 | SAXS scatter curves of extant and ancestral proteins at different protein concentrations

Supplementary Figure 3 | Cryo-EM data collection and processing schemes for SeCS

Supplementary Figure 4 | Cryo-EM data collection and processing schemes for Δ2-6 SeCS

Supplementary Figure 5 | Cryo-EM data collection and processing schemes H369R SeCS

Supplementary Figure 6 | Full phylogenetic tree of CS in Cyanobacteria

Supplementary Table 1 | Kinetic parameters for SeCS variants

Supplementary Table 2 | List and sequences of DNA sequences of proteins used in this study

Supplementary Table 3 | List and sequences of homology cassettes used for transformation of *S. elongatus* PCC7942

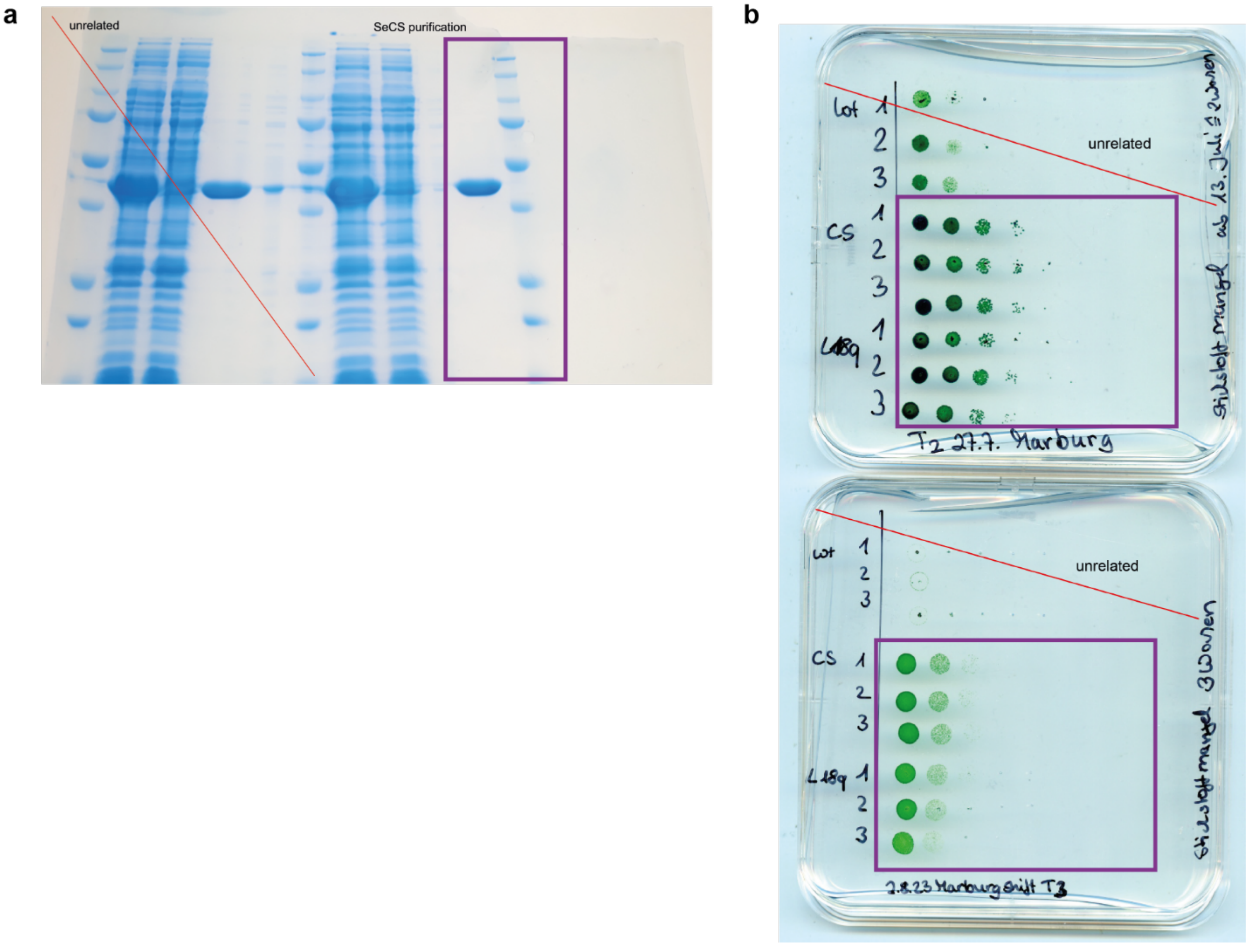

Supplementary Fig. 1 Uncropped images (**a**) SDS-PAGE of purified SeCS protein, see Extended Data Fig. 1a. (**b**) BG11-plates from survival assays of *S. elongatus* strains after extended nitrogen starvation, see Fig. 4f.

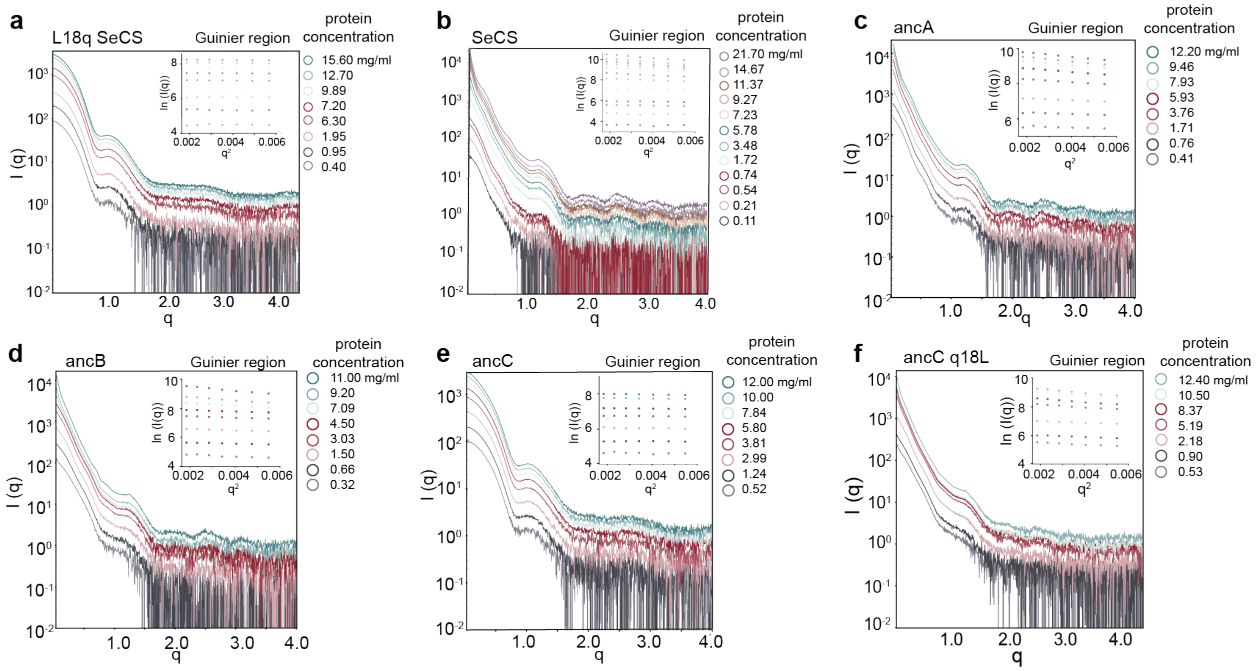

Supplementary Fig. 2 SAXS Raw scattering curves from SAXS measurements underlying the R_g measurements from (a) L18q SeCS, (b) WT SeCS, (c) ancA, (d) ancB, (e) ancC and (f) ancC q18L at indicated protein concentrations**.**

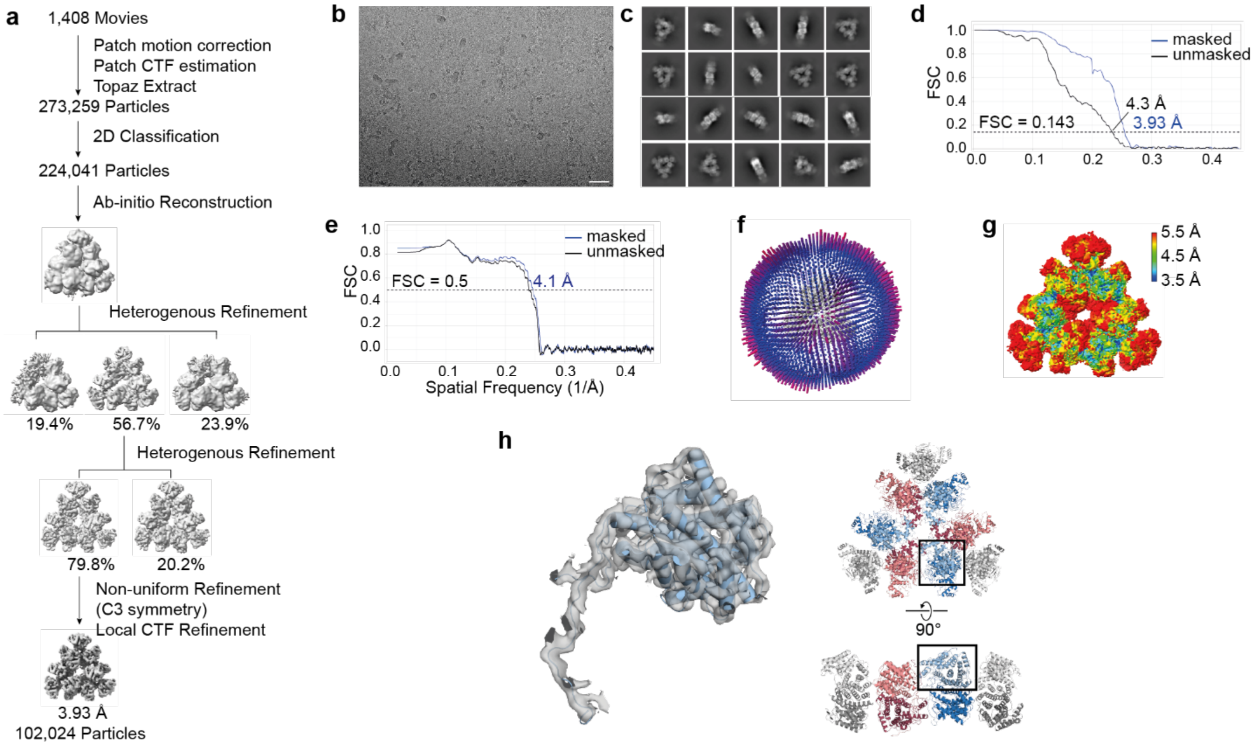

Supplementary Fig. 3 CryoEM processing workflow for the 18mer SeCS (**a**) An overview of the image processing procedure (see methods). (**b**) A representative micrograph of the SeCS (scale bar, 50 nm). 1,408 micrographs were collected it total. (**c**) Representative 2D class averages. (**d**) Fourier shell correlation (FSC) curves for the masked and unmasked reconstruction. (**e**) FSC curves for cross-validation between the summed modelling map and the final refined model with and without masking. (**f**) Euler angular distribution of particles used in the final 3D reconstruction. (**g**) Surface view of the local resolution of the reconstruction. **(h**) Cryo-EM density of a monomeric subunit forming the 18mer. The respective monomer is indicated in the full structure next to it.

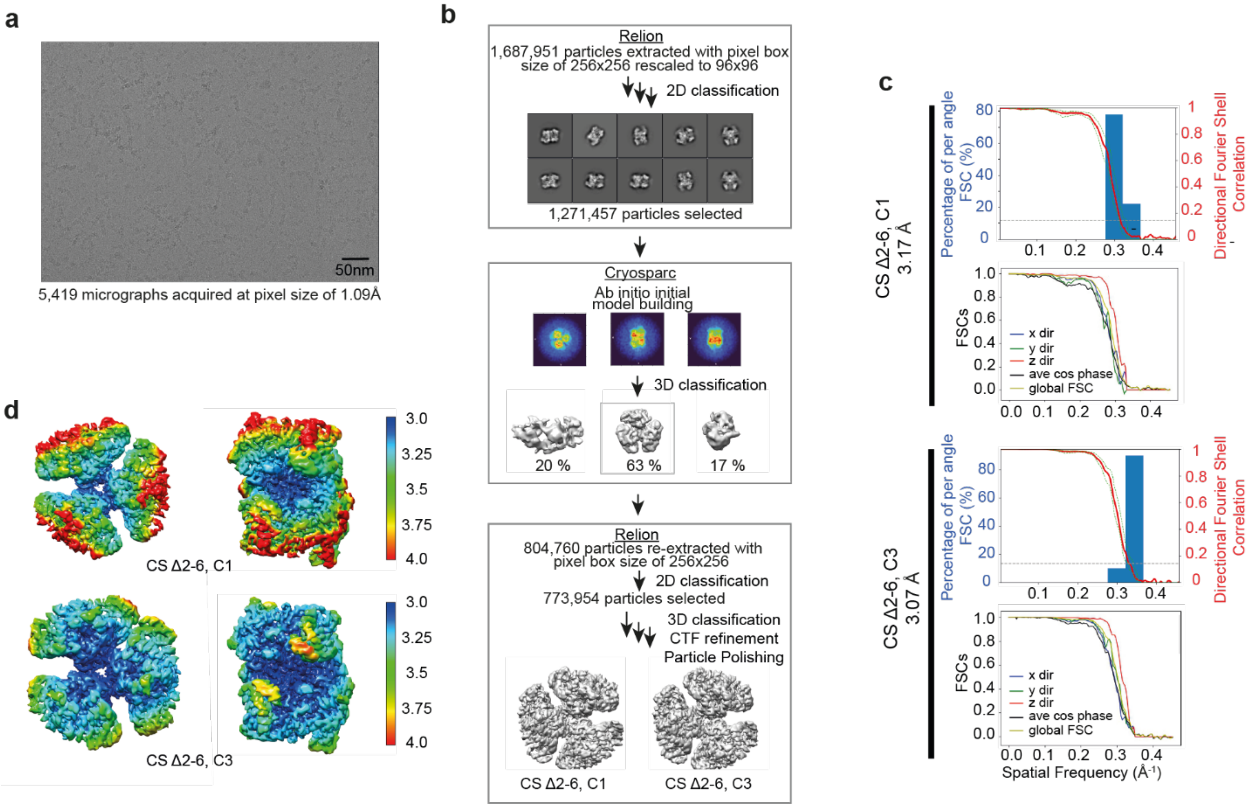

Supplementary Fig. 4 CryoEM processing workflow for the Δ2-6 SeCS (a) Example micrograph acquired on a K3 detector (Gatan, 5760x4092 pixel) showing particle distribution on Quantifoil 2/1 grid-holes. 5,419 micrographs were collected in total. **(b)** Schematic of the image processing workflow. crYOLO-picked particles were extracted and subjected to 2D classification with RELION. Selected classes were used for ab initio model building and 3D-classification in Cryosparc. Particles belonging to the 3D class reflecting 2D classification results were refined, subjected to Bayesian polishing and post-processed in RELION, once with C1 and once with C3 symmetry. **(c)** Plots showing the global and directional resolution for the densities obtained in (b), calculated using the "Remote 3DFSC Processing Server" web interface. For Δ2-6 C1 and C3 a sphericity score of 0.978 and 0.977 and a global resolution (FSC-threshold = 0.143) of 3.17 Å and of 3.07 Å was calculated, respectively. **(d)** Local resolution as calculated by RELION mapped on the refined densities (left: top view, right: side view).

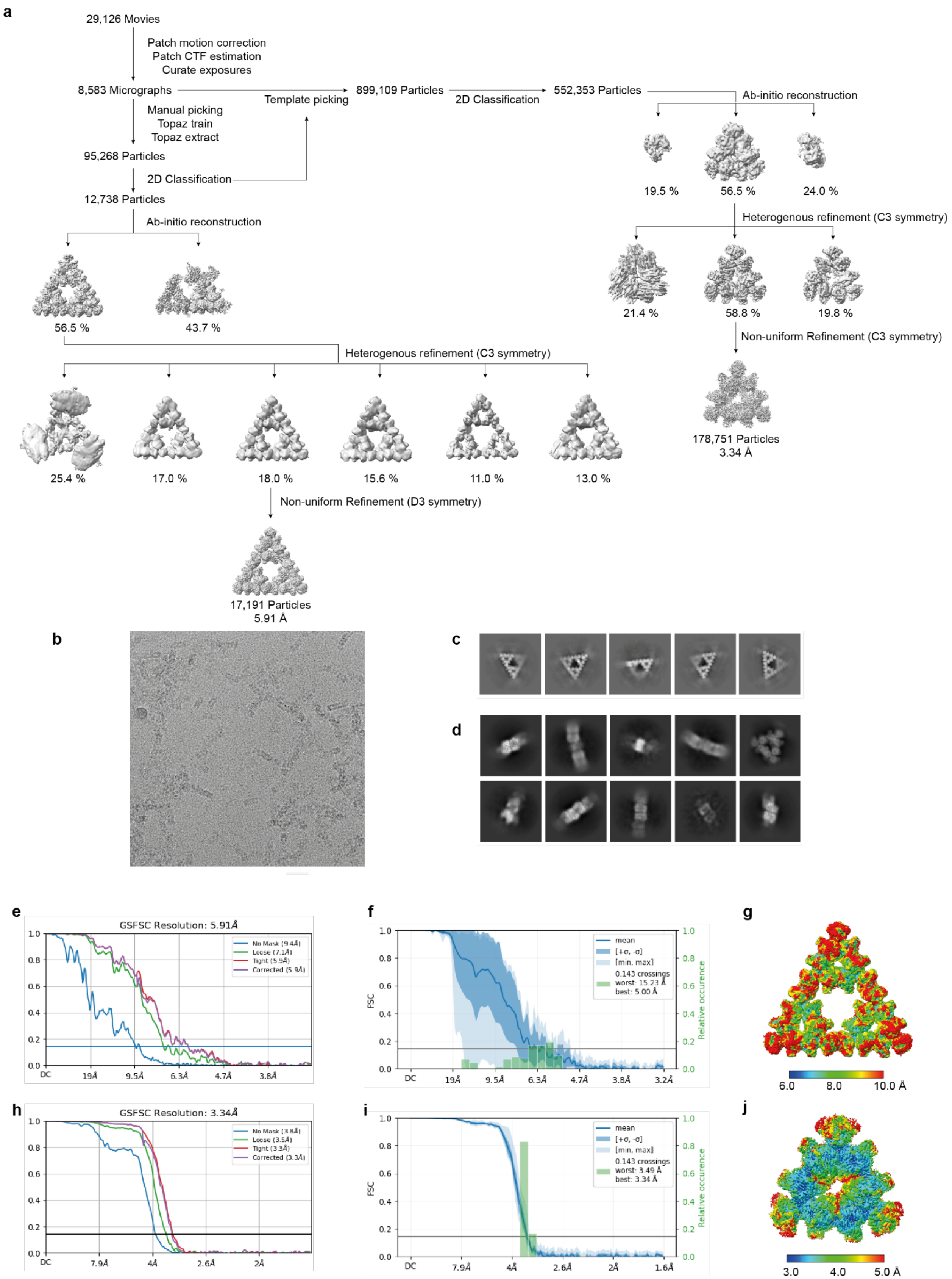

Supplementary Fig. 5 CryoEM processing workflow for H369R SeCS 18mer and 54mer

(**a**) An overview of the image processing procedure (see Methods). (**b**) A representative micrograph of the SeCS 54mer acquired from Thermo Scientific Krios G4 (scale bar, 50 nm). 29,126 micrographs were collected in total. (**c**) Representative 2D classes of 54mer and (**d**) 18mer. (**e**) Fourier shell correlation (FSC) curves for the reconstruction of 54mer before and after applying various masks. (**f**) Conical FSC curves of particles used in the final 54mer 3D reconstruction. (**g**) Density map of 54mer colored by local resolution. (**h**) FSC curve of 18mer before and after applying various masks. (**i**) Conical FSC curves of particles used in the final 18mer 3D reconstruction. (**j**) Density map of 18mer colored by local resolution.

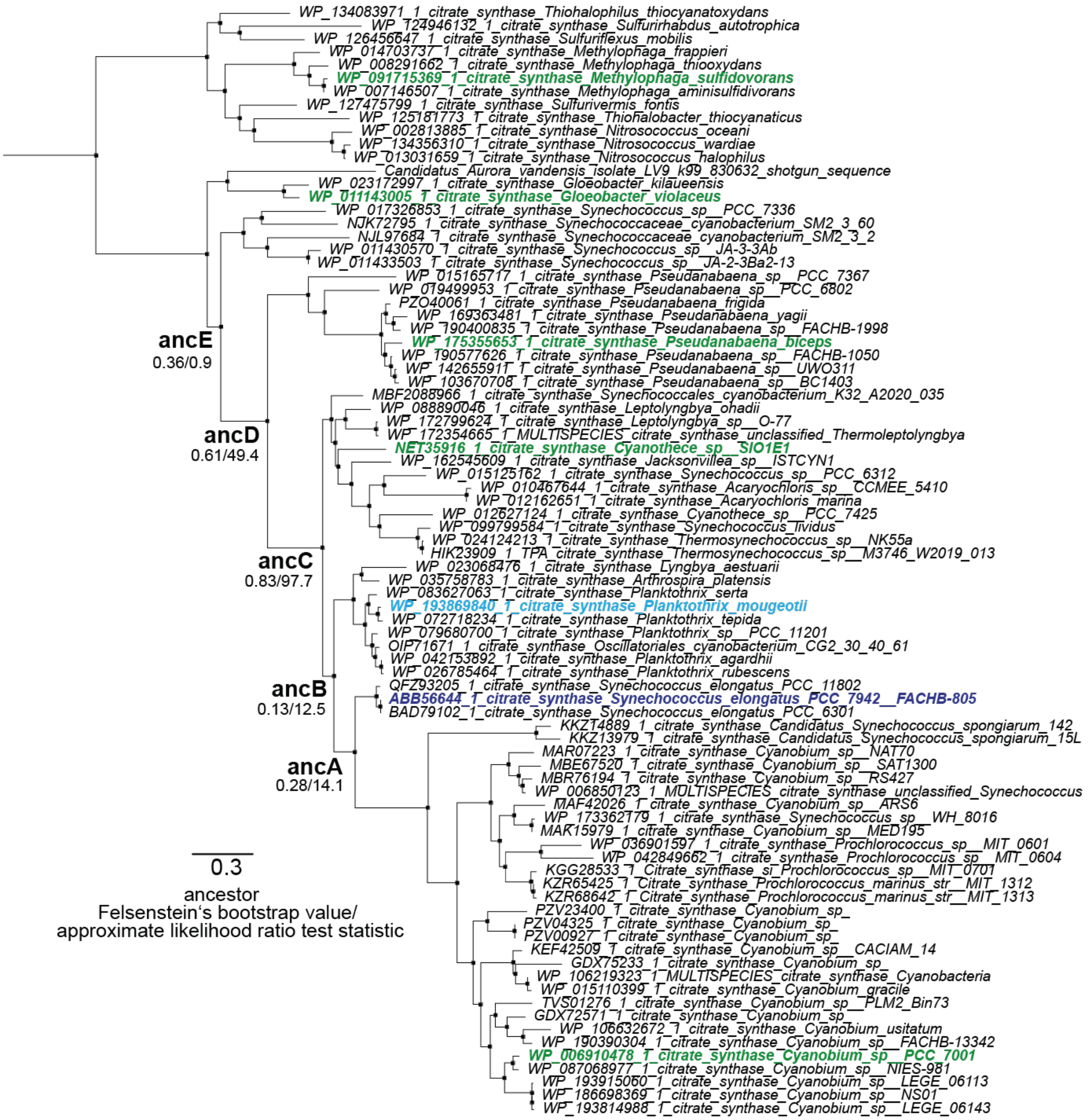

Supplementary Fig. 6 Phylogenetic tree of CS in Cyanobacteria Full tree with all sequences (organism name and NCBI-identifier) to infer the evolutionary history of SeCS. Extant sequences that were purified in vitro and analyzed regarding their assembly state are colored (blue and green). Resurrected nodes of the tree are indicated with corresponding Felsenstein's bootstrap values and approximate likelihood ratio test statistics.

Supplementary Table 1 Kinetic characterization for citrate synthase variants

	aceytlCoA		oxaloacetate	
	K_{cat} (s^{-1})	$K_m$acetyl-CoA (μM)	K_{cat} (s^{-1})	K_m oxaloacetate (μM)
**SeCS WT**	24.2 ± 1.3	91.8 ± 9.2	19.5 ± 1.7	65.6 ± 10.6
**SeCS L18Q**	21.7 ± 1.0	78.5 ± 6.2	20.5 ± 1.5	57.2 ± 7.8

Measurements were performed at 25 °C. N=3 biological replicates with 3 technical replicates each, errors = standard error of the mean

Supplementary Table 2 List of DNA sequences of proteins used in this study

Protein (Accession number)	DNA sequence
CS *S. elongatus* PCC 7942<br><br>GenBank: ABB56644.1	ATGACTGCCGTCAGCGAGTTTCGGCCTGGCCTAGAAGGCGTGCCCGCCACACTCTCGAGCATTAGCTTTGT<br>CGATGGCCAGCGCGGCGTCCTAGAGTATCGCGGCATCAGCATCGAGCAACTGGCGCAACAGAGCAGTTTT<br>CTGGAAACCGCCTACCTGTTGATTTGGGGCCATCTACCAACTCAGCAGGAATTGACCGAGTTCGAGCACGA<br>AATTCGCTACCACCGCCGCATCAAGTTCCGCATCCGGGACATGATGAAATGCTTCCCCGATAGCGGCCATC<br>CTATGGATGCCCTGCAGGCGAGCGCCGCAGCCCTCGGGTTGTTCTATTCGCGGCGCGCCTTGGATGATCC<br>CGAATACATTCGGGCGGCCGTTGTGCGTTTGCTAGCCAAAATTCCGACGATGGTGGCTGCCTTCCAGCTGA<br>TCCGCAAGGGTAACGACCCAATTCAGCCCCGCGATGAACTGGACTACGCCGCCAACTTTCTCTACATGCTG<br>ACGGAGCGCGAGCCCGATCCAGTCGCAGCTCGGATTTTTGATATTTGCCTCACCCTGCACGCCGAACATAC<br>GATCAACGCCTCGACCTTCTCGGCGATGGTCACAGCTTCGACCCTGACCGATCCCTACGCTGTCGTTGCTT<br>CTGCCGTTGGCACCTTGGCTGGCCCCCTCCATGGCGGCGCCAATGAAGAAGTGCTGGACATGCTGGAGGC<br>GATCGGTTCCGTCGAGAATGTTGAGCCCTACCTCGACCACTGCATTGCCACCAAAACGCGCATTATGGGCT<br>TTGGGCACCGTGTCTACAAAGTCAAGGATCCGCGGGCAGTCATTCTGCAAAATCTGGCCGAGCAACTGTTC<br>GATATCTTCGGCCATGATCCCTACTACGAAATCGCGGTCGCAGTCGAAAAGGCAGCAGCCGAGCGACTCAG<br>CCACAAGGGCATTTACCCCAACGTCGATTTCTACTCCGGCTTGGTCTATCGCAAGCTCGGTATTCCTAGCGA<br>TCTATTCACACCGGTGTTTGCGATCGCGCGGGTTGCGGGCTGGCTCGCCCACTGGAAAGAGCAGCTGAAC<br>GAAAATCGGATCTTCCGCCCCACTCAGATCTACACGGGCAGCCACAACCTCGACTACACCCCGATCGCCGA<br>TCGGGATTTGGCGATCGAATCTGAT
Δ2-5 CS *S. elongatus* PCC 7942	ATGGAGTTTCGGCCTGGCCTAGAAGGCGTGCCCGCCACACTCTCGAGCATTAGCTTTGTCGATGGCCAGCG<br>CGGCGTCCTAGAGTATCGCGGCATCAGCATCGAGCAACTGGCGCAACAGAGCAGTTTTCTGGAAACCGCCT<br>ACCTGTTGATTTGGGGCCATCTACCAACTCAGCAGGAATTGACCGAGTTCGAGCACGAAATTCGCTACCACC<br>GCCGCATCAAGTTCCGCATCCGGGACATGATGAAATGCTTCCCCGATAGCGGCCATCCTATGGATGCCCTG<br>CAGGCGAGCGCCGCAGCCCTCGGGTTGTTCTATTCGCGGCGCGCCTTGGATGATCCCGAATACATTCGGG<br>CGGCCGTTGTGCGTTTGCTAGCCAAAATTCCGACGATGGTGGCTGCCTTCCAGCTGATCCGCAAGGGTAAC<br>GACCCAATTCAGCCCCGCGATGAACTGGACTACGCCGCCAACTTTCTCTACATGCTGACGGAGCGCGAGCC<br>CGATCCAGTCGCAGCTCGGATTTTTGATATTTGCCTCACCCTGCACGCCGAACATACGATCAACGCCTCGAC<br>CTTCTCGGCGATGGTCACAGCTTCGACCCTGACCGATCCCTACGCTGTCGTTGCTTCTGCCGTTGGCACCT<br>TGGCTGGCCCCCTCCATGGCGGCGCCAATGAAGAAGTGCTGGACATGCTGGAGGCGATCGGTTCCGTCGA<br>GAATGTTGAGCCCTACCTCGACCACTGCATTGCCACCAAAACGCGCATTATGGGCTTTGGGCACCGTGTCT<br>ACAAAGTCAAGGATCCGCGGGCAGTCATTCTGCAAAATCTGGCCGAGCAACTGTTCGATATCTTCGGCCAT<br>GATCCCTACTACGAAATCGCGGTCGCAGTCGAAAAGGCAGCAGCCGAGCGACTCAGCCACAAGGGCATTT<br>ACCCCAACGTCGATTTCTACTCCGGCTTGGTCTATCGCAAGCTCGGTATTCCTAGCGATCTATTCACACCGG<br>TGTTTGCGATCGCGCGGGTTGCGGGCTGGCTCGCCCACTGGAAAGAGCAGCTGAACGAAAATCGGATCTT<br>CCGCCCCACTCAGATCTACACGGGCAGCCACAACCTCGACTACACCCCGATCGCCGATCGGGATTTGGCG<br>ATCGAATCTGAT
Δ2-6 CS *S. elongatus* PCC 7942	ATGTTTCGGCCTGGCCTAGAAGGCGTGCCCGCCACACTCTCGAGCATTAGCTTTGTCGATGGCCAGCGCG<br>GCGTCCTAGAGTATCGCGGCATCAGCATCGAGCAACTGGCGCAACAGAGCAGTTTTCTGGAAACCGCCTAC<br>CTGTTGATTTGGGGCCATCTACCAACTCAGCAGGAATTGACCGAGTTCGAGCACGAAATTCGCTACCACCG<br>CCGCATCAAGTTCCGCATCCGGGACATGATGAAATGCTTCCCCGATAGCGGCCATCCTATGGATGCCCTGC<br>AGGCGAGCGCCGCAGCCCTCGGGTTGTTCTATTCGCGGCGCGCCTTGGATGATCCCGAATACATTCGGGC<br>GGCCGTTGTGCGTTTGCTAGCCAAAATTCCGACGATGGTGGCTGCCTTCCAGCTGATCCGCAAGGGTAACG<br>ACCCAATTCAGCCCCGCGATGAACTGGACTACGCCGCCAACTTTCTCTACATGCTGACGGAGCGCGAGCCC<br>GATCCAGTCGCAGCTCGGATTTTTGATATTTGCCTCACCCTGCACGCCGAACATACGATCAACGCCTCGACC<br>TTCTCGGCGATGGTCACAGCTTCGACCCTGACCGATCCCTACGCTGTCGTTGCTTCTGCCGTTGGCACCTT<br>GGCTGGCCCCCTCCATGGCGGCGCCAATGAAGAAGTGCTGGACATGCTGGAGGCGATCGGTTCCGTCGAG<br>AATGTTGAGCCCTACCTCGACCACTGCATTGCCACCAAAACGCGCATTATGGGCTTTGGGCACCGTGTCTA<br>CAAAGTCAAGGATCCGCGGGCAGTCATTCTGCAAAATCTGGCCGAGCAACTGTTCGATATCTTCGGCCATG<br>ATCCCTACTACGAAATCGCGGTCGCAGTCGAAAAGGCAGCAGCCGAGCGACTCAGCCACAAGGGCATTTAC<br>CCCAACGTCGATTTCTACTCCGGCTTGGTCTATCGCAAGCTCGGTATTCCTAGCGATCTATTCACACCGGTG<br>TTTGCGATCGCGCGGGTTGCGGGCTGGCTCGCCCACTGGAAAGAGCAGCTGAACGAAAATCGGATCTTCC<br>GCCCCACTCAGATCTACACGGGCAGCCACAACCTCGACTACACCCCGATCGCCGATCGGGATTTGGCGATC<br>GAATCTGAT
E5A CS *S. elongatus* PCC 7942	ATGACTGCCGTCAGCGCATTTCGGCCTGGCCTAGAAGGCGTGCCCGCCACACTCTCGAGCATTAGCTTTGT<br>CGATGGCCAGCGCGGCGTCCTAGAGTATCGCGGCATCAGCATCGAGCAACTGGCGCAACAGAGCAGTTTT<br>CTGGAAACCGCCTACCTGTTGATTTGGGGCCATCTACCAACTCAGCAGGAATTGACCGAGTTCGAGCACGA<br>AATTCGCTACCACCGCCGCATCAAGTTCCGCATCCGGGACATGATGAAATGCTTCCCCGATAGCGGCCATC<br>CTATGGATGCCCTGCAGGCGAGCGCCGCAGCCCTCGGGTTGTTCTATTCGCGGCGCGCCTTGGATGATCC<br>CGAATACATTCGGGCGGCCGTTGTGCGTTTGCTAGCCAAAATTCCGACGATGGTGGCTGCCTTCCAGCTGA<br>TCCGCAAGGGTAACGACCCAATTCAGCCCCGCGATGAACTGGACTACGCCGCCAACTTTCTCTACATGCTG<br>ACGGAGCGCGAGCCCGATCCAGTCGCAGCTCGGATTTTTGATATTTGCCTCACCCTGCACGCCGAACATAC<br>GATCAACGCCTCGACCTTCTCGGCGATGGTCACAGCTTCGACCCTGACCGATCCCTACGCTGTCGTTGCTT<br>CTGCCGTTGGCACCTTGGCTGGCCCCCTCCATGGCGGCGCCAATGAAGAAGTGCTGGACATGCTGGAGGC<br>GATCGGTTCCGTCGAGAATGTTGAGCCCTACCTCGACCACTGCATTGCCACCAAAACGCGCATTATGGGCT<br>TTGGGCACCGTGTCTACAAAGTCAAGGATCCGCGGGCAGTCATTCTGCAAAATCTGGCCGAGCAACTGTTC<br>GATATCTTCGGCCATGATCCCTACTACGAAATCGCGGTCGCAGTCGAAAAGGCAGCAGCCGAGCGACTCAG<br>CCACAAGGGCATTTACCCCAACGTCGATTTCTACTCCGGCTTGGTCTATCGCAAGCTCGGTATTCCTAGCGA<br>TCTATTCACACCGGTGTTTGCGATCGCGCGGGTTGCGGGCTGGCTCGCCCACTGGAAAGAGCAGCTGAAC<br>GAAAATCGGATCTTCCGCCCCACTCAGATCTACACGGGCAGCCACAACCTCGACTACACCCCGATCGCCGA<br>TCGGGATTTGGCGATCGAATCTGAT

L18Q CS *S. elongatus* PCC 7942	ATGACTGCCGTCAGCGAGTTTCGGCCTGGCCTAGAAGGCGTGCCCGCCACACAGTCGAGCATTAGCTTTGT<br>CGATGGCCAGCGCGGCGTCCTAGAGTATCGCGGCATCAGCATCGAGCAACTGGCGCAACAGAGCAGTTTT<br>CTGGAAACCGCCTACCTGTTGATTTGGGGCCATCTACCAACTCAGCAGGAATTGACCGAGTTCGAGCACGA<br>AATTCGCTACCACCGCCGCATCAAGTTCCGCATCCGGGACATGATGAAATGCTTCCCCGATAGCGGCCATC<br>CTATGGATGCCCTGCAGGCGAGCGCCGCAGCCCTCGGGTTGTTCTATTCGCGGCGCGCCTTGGATGATCC<br>CGAATACATTCGGGCGGCCGTTGTGCGTTTGCTAGCCAAAATTCCGACGATGGTGGCTGCCTTCCAGCTGA<br>TCCGCAAGGGTAACGACCCAATTCAGCCCCGCGATGAACTGGACTACGCCGCCAACTTTCTCTACATGCTG<br>ACGGAGCGCGAGCCCGATCCAGTCGCAGCTCGGATTTTTGATATTTGCCTCACCCTGCACGCCGAACATAC<br>GATCAACGCCTCGACCTTCTCGGCGATGGTCACAGCTTCGACCCTGACCGATCCCTACGCTGTCGTTGCTT<br>CTGCCGTTGGCACCTTGGCTGGCCCCCTCCATGGCGGCGCCAATGAAGAAGTGCTGGACATGCTGGAGGC<br>GATCGGTTCCGTCGAGAATGTTGAGCCCTACCTCGACCACTGCATTGCCACCAAAACGCGCATTATGGGCT<br>TTGGGCACCGTGTCTACAAAGTCAAGGATCCGCGGGCAGTCATTCTGCAAAATCTGGCCGAGCAACTGTTC<br>GATATCTTCGGCCATGATCCCTACTACGAAATCGCGGTCGCAGTCGAAAAGGCAGCAGCCGAGCGACTCAG<br>CCACAAGGGCATTTACCCCAACGTCGATTTCTACTCCGGCTTGGTCTATCGCAAGCTCGGTATTCCTAGCGA<br>TCTATTCACACCGGTGTTTGCGATCGCGCGGGTTGCGGGCTGGCTCGCCCACTGGAAAGAGCAGCTGAAC<br>GAAAATCGGATCTTCCGCCCCACTCAGATCTACACGGGCAGCCACAACCTCGACTACACCCCGATCGCCGA<br>TCGGGATTTGGCGATCGAATCTGAT
H369R CS *S. elongatus* PCC 7942	ATGACTGCCGTCAGCGAGTTTCGGCCTGGCCTAGAAGGCGTGCCCGCCACACTCTCGAGCATTAGCTTTGT<br>CGATGGCCAGCGCGGCGTCCTAGAGTATCGCGGCATCAGCATCGAGCAACTGGCGCAACAGAGCAGTTTT<br>CTGGAAACCGCCTACCTGTTGATTTGGGGCCATCTACCAACTCAGCAGGAATTGACCGAGTTCGAGCACGA<br>AATTCGCTACCACCGCCGCATCAAGTTCCGCATCCGGGACATGATGAAATGCTTCCCCGATAGCGGCCATC<br>CTATGGATGCCCTGCAGGCGAGCGCCGCAGCCCTCGGGTTGTTCTATTCGCGGCGCGCCTTGGATGATCC<br>CGAATACATTCGGGCGGCCGTTGTGCGTTTGCTAGCCAAAATTCCGACGATGGTGGCTGCCTTCCAGCTGA<br>TCCGCAAGGGTAACGACCCAATTCAGCCCCGCGATGAACTGGACTACGCCGCCAACTTTCTCTACATGCTG<br>ACGGAGCGCGAGCCCGATCCAGTCGCAGCTCGGATTTTTGATATTTGCCTCACCCTGCACGCCGAACATAC<br>GATCAACGCCTCGACCTTCTCGGCGATGGTCACAGCTTCGACCCTGACCGATCCCTACGCTGTCGTTGCTT<br>CTGCCGTTGGCACCTTGGCTGGCCCCCTCCATGGCGGCGCCAATGAAGAAGTGCTGGACATGCTGGAGGC<br>GATCGGTTCCGTCGAGAATGTTGAGCCCTACCTCGACCACTGCATTGCCACCAAAACGCGCATTATGGGCT<br>TTGGGCACCGTGTCTACAAAGTCAAGGATCCGCGGGCAGTCATTCTGCAAAATCTGGCCGAGCAACTGTTC<br>GATATCTTCGGCCATGATCCCTACTACGAAATCGCGGTCGCAGTCGAAAAGGCAGCAGCCGAGCGACTCAG<br>CCACAAGGGCATTTACCCCAACGTCGATTTCTACTCCGGCTTGGTCTATCGCAAGCTCGGTATTCCTAGCGA<br>TCTATTCACACCGGTGTTTGCGATCGCGCGGGTTGCGGGCTGGCTCGCCCACTGGAAAGAGCAGCTGAAC<br>GAAAATCGGATCTTCCGCCCCACTCAGATCTACACGGGCAGCCGCAACCTCGACTACACCCCGATCGCCGA<br>TCGGGATTTGGCGATCGAATCTGAT
Cys4 CS *S. elongatus* PCC 7942	ATGACTGCCTGCAGCGAGTTTCGGCCTGGCCTAGAAGGCGTGCCCGCCACACTCTCGAGCATTAGCTTTGT<br>CGATGGCCAGCGCGGCGTCCTAGAGTATCGCGGCATCAGCATCGAGCAACTGGCGCAACAGAGCAGTTTT<br>CTGGAAACCGCCTACCTGTTGATTTGGGGCCATCTACCAACTCAGCAGGAATTGACCGAGTTCGAGCACGA<br>AATTCGCTACCACCGCCGCATCAAGTTCCGCATCCGGGACATGATGAAATGCTTCCCCGATAGCGGCCATC<br>CTATGGATGCCCTGCAGGCGAGCGCCGCAGCCCTCGGGTTGTTCTATTCGCGGCGCGCCTTGGATGATCC<br>CGAATACATTCGGGCGGCCGTTGTGCGTTTGCTAGCCAAAATTCCGACGATGGTGGCTGCCTTCCAGCTGA<br>TCCGCAAGGGTAACGACCCAATTCAGCCCCGCGATGAACTGGACTACGCCGCCAACTTTCTCTACATGCTG<br>ACGGAGCGCGAGCCCGATCCAGTCGCAGCTCGGATTTTTGATATTTGCCTCACCCTGCACGCCGAACATAC<br>GATCAACGCCTCGACCTTCTCGGCGATGGTCACAGCTTCGACCCTGACCGATCCCTACGCTGTCGTTGCTT<br>CTGCCGTTGGCACCTTGGCTGGCCCCCTCCATGGCGGCGCCAATGAAGAAGTGCTGGACATGCTGGAGGC<br>GATCGGTTCCGTCGAGAATGTTGAGCCCTACCTCGACCACTGCATTGCCACCAAAACGCGCATTATGGGCT<br>TTGGGCACCGTGTCTACAAAGTCAAGGATCCGCGGGCAGTCATTCTGCAAAATCTGGCCGAGCAACTGTTC<br>GATATCTTCGGCCATGATCCCTACTACGAAATCGCGGTCGCAGTCGAAAAGGCAGCAGCCGAGCGACTCAG<br>CCACAAGGGCATTTACCCCAACGTCGATTTCTACTCCGGCTTGGTCTATCGCAAGCTCGGTATTCCTAGCGA<br>TCTATTCACACCGGTGTTTGCGATCGCGCGGGTTGCGGGCTGGCTCGCCCACTGGAAAGAGCAGCTGAAC<br>GAAAATCGGATCTTCCGCCCCACTCAGATCTACACGGGCAGCCACAACCTCGACTACACCCCGATCGCCGA<br>TCGGGATTTGGCGATCGAATCTGAT
CS *Synechocystis* PCC 6803<br><br>NCBI Reference Sequence: WP_010873488 .1	ATGAATTATATGATGACTGATAACGAAGTGTTTAAAGAAGGCCTAGCCGGAGTCCCCGCCGCTAAATCGAGG<br>GTGAGCCATGTGGATGGCACCGACGGTATTTTGGAGTACCGGGGCATTCGCATCGAAGAATTAGCCAAATC<br>CAGTAGTTTTATCGAAGTAGCCTATCTGCTCATCTGGGGTAAATTGCCCACCCAGGCAGAGATCGAAGAGTT<br>TGAGTACGAAATTCGCACCCATCGACGCATTAAGTACCACATCCGGGACATGATGAAATGTTTTCCCGAAAC<br>AGGGCACCCCATGGATGCCTTGCAAACTTCAGCGGCGGCCTTGGGATTGTTCTATGCTCGACGGGCCTTGG<br>ATGACCCCAAATATATCCGGGCGGCGGTGGTGCGTCTGTTAGCCAAAATCCCCACCATGGTGGCAGCTTTC<br>CACATGATCCGGGAGGGTAACGATCCCATTCAGCCCAATGATAAATTGGATTACGCTTCCAACTTCCTTTAC<br>ATGCTGACGGAGAAGGAGCCAGACCCCTTTGCCGCCAAAGTGTTTGATGTGTGTTTGACCCTCCATGCTGA<br>GCACACCATGAATGCGTCCACCTTTTCGGCCCGGGTAACGGCTTCTACTCTCACGGATCCCTATGCAGTGG<br>TTGCCTCGGCGGTGGGGACTTTGGCGGGGCCGCTCCACGGGGGAGCCAACGAAGAAGTGCTAAATATGCT<br>TGAAGAAATTGGCTCAGTGGAAAATGTCCGCCCCTACGTGGAAAAATGCCTGGCCAACAAACAGCGCATCA<br>TGGGCTTTGGCCACCGAGTTTATAAAGTCAAAGACCCCCGGGCAATTATTTTGCAGGATTTGGCTGAACAGT<br>TATTTGCCAAAATGGGCCACGACGAATATTACGAAATCGCAGTGGAGTTGGAAAAAGTAGTGGAAGAATACG<br>TGGGTCAAAAGGGCATTTACCCCAATGTGGACTTCTATTCCGGTTTGGTTTACCGCAAGCTAGACATCCCCG<br>CCGATCTGTTTACGCCCCTATTTGCGATCGCCAGGGTGGCGGGTTGGTTGGCCCACTGGAAGGAACAATTA<br>TCAGTCAATAAAATTTACCGTCCTACCCAAATTTACATCGGTGACCATAATTTATCTTATGTTCCCATGACAGA<br>ACGGGTAGTTTCCGTGGCCCGCAATGAAGACCCCAATGCGATTATT
ancA	ATGACTGCCGTATCCGAGTTTCGTCCAGGGCTTGAGGGAGTACCTGCAACTTTGAGCTCGATCAGTTTTGTG<br>GACGGGCAACGCGGTGTACTTGAATATCGTGGGATTTCCATCGAGCAACTGGCGCAACATTCTTCTTTTCTT<br>GAGACTGCCTATTTACTGATCTGGGGCCATCTTCCGACTCAAGAAGAGCTTACGGAGTTCGAGCATGAGATC<br>CGTTATCATCGTCGTATTAAATTTCGCATCCGTGACATGATGAAATGTTTTCCTGAGTCAGGGCACCCGATG<br>GACGCTTTACAGGCTTCTGCTGCCGCTTTGGGACTTTTCTACTCTCGCCGCGCTTTGGACGACCCAGAATAT<br>ATTCGTGCAGCAGTTGTTCGTCTGTTAGCTAAAATCCCAACAATGGTCGCGGCATTCCAACTTATTCGTAAAG<br>GAAATGATCCGATCCAGCCTCGCGACGACCTGGATTATGCCGCTAATTTCTTATACATGCTTACAGAACGCG<br>AGCCGGACCCTCTTGCCGCACGCATCTTTGACGTCTGTCTTACCCTTCACGCTGAACACACTATTAACGCAT<br>CTACCTTTTCAGCCATGGTTACAGCATCCACTCTTACAGACCCTTATGCCGTAGTGGCTTCAGCCGTCGGGA<br>CGCTTGCCGGCCCATTGCACGGTGGGGCTAATGAGGAAGTTCTTGACATGCTGGAAGAGATTGGTAGCGTC<br>GAAAATGTTGAGCCGTATTTAGATCATTGCATTGCAAAGAAAACCAAGATTATGGGCTTCGGTCACCGCGTA<br>TATAAAGTAAAAGATCCACGTGCTGTAATCTTGCAGAACCTTGCCGAGCAACTGTTTGATAAATTTGGTCACG<br>ATCCGTACTATGAAATTGCCGTAGCCTTAGAGAAGGCCGCGGCCGAGCGTCTGGGACATAAGGGCATCTAT

	CCAAACGTTGATTTTTATTCTGGGCTTGTGTATCGCAAATTAGGAATCCCGTCCGACCTTTTTACTCCGGTCT<br>TCGCAATCGCGCGTGTTGCCGGGTGGCTGGCACACTGGAAAGAGCAGTTGAATGAGAACCGTATTTTCCGC<br>CCTACACAAATCTACACAGGTAGCCATAATCTGAATTATGTCCCGATCGAGGAACGCGATTTGGCGATCGAA<br>TCTGAT
ancA L18q	ATGACTGCCGTATCCGAGTTTCGTCCAGGGCTTGAGGGAGTACCTGCAACTCAGAGCTCGATCAGTTTTGT<br>GGACGGGCAACGCGGTGTACTTGAATATCGTGGGATTTCCATCGAGCAACTGGCGCAACATTCTTCTTTTCT<br>TGAGACTGCCTATTTACTGATCTGGGGCCATCTTCCGACTCAAGAAGAGCTTACGGAGTTCGAGCATGAGAT<br>CCGTTATCATCGTCGTATTAAATTTCGCATCCGTGACATGATGAAATGTTTTCCTGAGTCAGGGCACCCGAT<br>GGACGCTTTACAGGCTTCTGCTGCCGCTTTGGGACTTTTCTACTCTCGCCGCGCTTTGGACGACCCAGAATA<br>TATTCGTGCAGCAGTTGTTCGTCTGTTAGCTAAAATCCCAACAATGGTCGCGGCATTCCAACTTATTCGTAAA<br>GGAAATGATCCGATCCAGCCTCGCGACGACCTGGATTATGCCGCTAATTTCTTATACATGCTTACAGAACGC<br>GAGCCGGACCCTCTTGCCGCACGCATCTTTGACGTCTGTCTTACCCTTCACGCTGAACACACTATTAACGCA<br>TCTACCTTTTCAGCCATGGTTACAGCATCCACTCTTACAGACCCTTATGCCGTAGTGGCTTCAGCCGTCGGG<br>ACGCTTGCCGGCCCATTGCACGGTGGGGCTAATGAGGAAGTTCTTGACATGCTGGAAGAGATTGGTAGCGT<br>CGAAAATGTTGAGCCGTATTTAGATCATTGCATTGCAAAGAAAACCAAGATTATGGGCTTCGGTCACCGCGT<br>ATATAAAGTAAAAGATCCACGTGCTGTAATCTTGCAGAACCTTGCCGAGCAACTGTTTGATAAATTTGGTCAC<br>GATCCGTACTATGAAATTGCCGTAGCCTTAGAGAAGGCCGCGGCCGAGCGTCTGGGACATAAGGGCATCTA<br>TCCAAACGTTGATTTTTATTCTGGGCTTGTGTATCGCAAATTAGGAATCCCGTCCGACCTTTTTACTCCGGTC<br>TTCGCAATCGCGCGTGTTGCCGGGTGGCTGGCACACTGGAAAGAGCAGTTGAATGAGAACCGTATTTTCCG<br>CCCTACACAAATCTACACAGGTAGCCATAATCTGAATTATGTCCCGATCGAGGAACGCGATTTGGCGATCGA<br>ATCTGAT
AltaII ancA	ATGACAGCGGTTTCGGAATTCCGTCCAGGTCTTGAGGGAGTTCCAGCGACGCAGTCATCCATTTCTTACGTA<br>GATGGACAAAAGGGTATCTTAGAATATCGCGGGATCAGCATCGAACAGCTGGCACAGCAATCCTCATTCCTT<br>GAAACCGCCTACCTTTTAATTTGGGGTAAACTTCCAACGCAACAGGAGCTGGCAGAGTTTGAACACGAAATT<br>CGTTACCACCGTCGCATCAAGTTCCGTATCCGTGATATGATGAAATGTTTCCCCGATTCTGGGCACCCCATG<br>GATGCACTTCAGGCTTCAGCCGCCGCGCTTGGCCTTTTTTACAGTCGTCGCGCTCTTGACGACCCGGAATA<br>CATTCGTGCAGCCGTTGTGCGCCTGCTGGCCAAGATCCCCACTATGGTTGCCGCATTTCAGTTAATCCGCA<br>AGGGTAATGATCCGATCCAGCCACGTGATGATCTGGATTACGCTGCGAACTTTCTTTACATGTTAACAGAGC<br>GTGAGCCAGATCCTCTGGCTGCCCGTATTTTCGATGTTTGTTTAACTTTGCACGCAGAACACACTATCAATG<br>CAAGTACCTTTTCAGCTATGGTTACGGCCTCTACCCTGACCGATCCATATGCTGTCGTAGCGTCTGCAGTTG<br>GGACGCTGGCGGGACCTCTTCATGGGGGCGCAAATGAAGAGGTACTTACAATGTTGGAAGAGATTGGAAGT<br>GTCGAGAACGTAGAGCCGTACTTGGACCACTGCATTGCACGCAAGGCGCGCATCATGGGGTTCGGTCACC<br>GCGTGTACAAAGTAAAAGACCCTCGTGCTGTCATTCTTCAGAACCTGGCTGAGCAACTTTTTGAGCGTTTTG<br>GTCACGACCCATATTACGACATCGCGTTAGCATTGGAACGTGCAGTCGCCGAACGCCTGGGCCACAAGGG<br>GATTTATCCAAACGTCGACTTTTACTCAGGACTTGTGTATCGCAAACTTGGTATCCCTTCAGACTTATTTACA<br>CCCGTTTTCGCTATCGCACGTGTTGCAGGGTGGCTTGCCCATTGGAAGGAACAGTTATCAGAAAATCGCATT<br>TTTCGCCCCACGCAAATTTATACAGGTAGTCACAACATGGATTATACACCAATTGAGGAGCGTGATTTGGCG<br>ATCGAATCTGAT
AltaII ancA L18q	ATGACAGCGGTTTCGGAATTCCGTCCAGGTCTTGAGGGAGTTCCAGCGACGCTCTCATCCATTTCTTACGTA<br>GATGGACAAAAGGGTATCTTAGAATATCGCGGGATCAGCATCGAACAGCTGGCACAGCAATCCTCATTCCTT<br>GAAACCGCCTACCTTTTAATTTGGGGTAAACTTCCAACGCAACAGGAGCTGGCAGAGTTTGAACACGAAATT<br>CGTTACCACCGTCGCATCAAGTTCCGTATCCGTGATATGATGAAATGTTTCCCCGATTCTGGGCACCCCATG<br>GATGCACTTCAGGCTTCAGCCGCCGCGCTTGGCCTTTTTTACAGTCGTCGCGCTCTTGACGACCCGGAATA<br>CATTCGTGCAGCCGTTGTGCGCCTGCTGGCCAAGATCCCCACTATGGTTGCCGCATTTCAGTTAATCCGCA<br>AGGGTAATGATCCGATCCAGCCACGTGATGATCTGGATTACGCTGCGAACTTTCTTTACATGTTAACAGAGC<br>GTGAGCCAGATCCTCTGGCTGCCCGTATTTTCGATGTTTGTTTAACTTTGCACGCAGAACACACTATCAATG<br>CAAGTACCTTTTCAGCTATGGTTACGGCCTCTACCCTGACCGATCCATATGCTGTCGTAGCGTCTGCAGTTG<br>GGACGCTGGCGGGACCTCTTCATGGGGGCGCAAATGAAGAGGTACTTACAATGTTGGAAGAGATTGGAAGT<br>GTCGAGAACGTAGAGCCGTACTTGGACCACTGCATTGCACGCAAGGCGCGCATCATGGGGTTCGGTCACC<br>GCGTGTACAAAGTAAAAGACCCTCGTGCTGTCATTCTTCAGAACCTGGCTGAGCAACTTTTTGAGCGTTTTG<br>GTCACGACCCATATTACGACATCGCGTTAGCATTGGAACGTGCAGTCGCCGAACGCCTGGGCCACAAGGG<br>GATTTATCCAAACGTCGACTTTTACTCAGGACTTGTGTATCGCAAACTTGGTATCCCTTCAGACTTATTTACA<br>CCCGTTTTCGCTATCGCACGTGTTGCAGGGTGGCTTGCCCATTGGAAGGAACAGTTATCAGAAAATCGCATT<br>TTTCGCCCCACGCAAATTTATACAGGTAGTCACAACATGGATTATACACCAATTGAGGAGCGTGATTTGGCG<br>ATCGAATCTGAT
ancB	ATGACGGCTGTCTCCGAGTTTAAGCCTGGACTTGAGGGTGTGCCCGCTACTCTGAGTTCTATTTCCTTCGTC<br>GACGGCCAGCGCGGGATTCTGGAATACCGTGGGATTAGTATTGAGCAATTAGCCCAACATTCGACATTTCTT<br>GAGACAGCGTATTTACTGATTTGGGGTAAACTTCCCACCCAAGAGGAATTAGCAGAATTCGAACATGAAATT<br>CGCTACCATCGCCGTATCAAGTACCGTATTCGTGATATGATGAAGTGCTTCCCAGAGTCAGGTCACCCAATG<br>GATGCTTTGCAAGCCTCAGCAGCGGCCTTGGGGTTGTTCTATTCGCGTCGTGCGCTGGACGACCCCGAGTA<br>CATCCGTGCGGCAGTTGTGCGTTTACTGGCCAAGATTCCGACAATGGTTGCAGCATTCCAACTTATGCGCAA<br>AGGAAACGACCCGGTACAACCGCGTGATGATCTGGATTATGCGGCTAACTTCTTGTACATGTTAAACGAACG<br>TGAGCCAGATCCGCTGGCCGCACGCATCTTCGACGTATGCCTTACGCTGCATGCGGAACATACTATCAACG<br>CCTCCACGTTCTCGGCAATGGTAACGGCGTCTACTTTAACTGACCCTTACGCAGTAGTAGCATCGGCTGTAG<br>GGACGTTAGCCGGCCCGTTACATGGAGGTGCAAACGAGGAGGTTTTGACAATGTTGGAGGAGATTGGCAG<br>TGTTGAGAACGTCCGTCCGTACTTAGACCATTGTATCGCGAAAAAGGCCAAGATCATGGGGTTCGGACACC<br>GCGTGTATAAAGTAAAGGATCCTCGTGCGACAATTTTACAAAATTTAGCAGAGCAGTTATTTGAGAAATTTGG<br>TCACGATCAGTATTATGAAATCGCACTGGAATTAGAGCGTGTAGTCGCTGAACGCCTTGGTCATAAAGGGAT<br>TTATCCGAACGTCGATTTTTATTCTGGATTGGTATACCGTAAATTAGGCATTCCATCGGATTTATTCACTCCG<br>GTATTTGCCATCGCACGCGTGGCAGGTTGGTTGGCCCACTGGAAAGAACAATTGTCCGAAAATCGCATCTT<br>CCGTCCAACCCAAATTTATACTGGATCACATAATATGACTTACGTGCCAATCGAAGAACGTGATTTGGCGAT<br>CGAATCTGAT
ancB k8R	ATGACGGCTGTCTCCGAGTTTCGTCCTGGACTTGAGGGTGTGCCCGCTACTCTGAGTTCTATTTCCTTCGTC<br>GACGGCCAGCGCGGGATTCTGGAATACCGTGGGATTAGTATTGAGCAATTAGCCCAACATTCGACATTTCTT<br>GAGACAGCGTATTTACTGATTTGGGGTAAACTTCCCACCCAAGAGGAATTAGCAGAATTCGAACATGAAATT<br>CGCTACCATCGCCGTATCAAGTACCGTATTCGTGATATGATGAAGTGCTTCCCAGAGTCAGGTCACCCAATG<br>GATGCTTTGCAAGCCTCAGCAGCGGCCTTGGGGTTGTTCTATTCGCGTCGTGCGCTGGACGACCCCGAGTA<br>CATCCGTGCGGCAGTTGTGCGTTTACTGGCCAAGATTCCGACAATGGTTGCAGCATTCCAACTTATGCGCAA<br>AGGAAACGACCCGGTACAACCGCGTGATGATCTGGATTATGCGGCTAACTTCTTGTACATGTTAAACGAACG<br>TGAGCCAGATCCGCTGGCCGCACGCATCTTCGACGTATGCCTTACGCTGCATGCGGAACATACTATCAACG<br>CCTCCACGTTCTCGGCAATGGTAACGGCGTCTACTTTAACTGACCCTTACGCAGTAGTAGCATCGGCTGTAG

	GGACGTTAGCCGGCCCGTTACATGGAGGTGCAAACGAGGAGGTTTTGACAATGTTGGAGGAGATTGGCAG<br>TGTTGAGAACGTCCGTCCGTACTTAGACCATTGTATCGCGAAAAAGGCCAAGATCATGGGGTTCGGACACC<br>GCGTGTATAAAGTAAAGGATCCTCGTGCGACAATTTTACAAAATTTAGCAGAGCAGTTATTTGAGAAATTTGG<br>TCACGATCAGTATTATGAAATCGCACTGGAATTAGAGCGTGTAGTCGCTGAACGCCTTGGTCATAAAGGGAT<br>TTATCCGAACGTCGATTTTTATTCTGGATTGGTATACCGTAAATTAGGCATTCCATCGGATTTATTCACTCCG<br>GTATTTGCCATCGCACGCGTGGCAGGTTGGTTGGCCCACTGGAAAGAACAATTGTCCGAAAATCGCATCTT<br>CCGTCCAACCCAAATTTATACTGGATCACATAATATGACTTACGTGCCAATCGAAGAACGTGATTTGGCGAT<br>CGAATCTGAT
ancB y80F	ATGACGGCTGTCTCCGAGTTTAAGCCTGGACTTGAGGGTGTGCCCGCTACTCTGAGTTCTATTTCCTTCGTC<br>GACGGCCAGCGCGGGATTCTGGAATACCGTGGGATTAGTATTGAGCAATTAGCCCAACATTCGACATTTCTT<br>GAGACAGCGTATTTACTGATTTGGGGTAAACTTCCCACCCAAGAGGAATTAGCAGAATTCGAACATGAAATT<br>CGCTACCATCGCCGTATCAAGTTCCGTATTCGTGATATGATGAAGTGCTTCCCAGAGTCAGGTCACCCAATG<br>GATGCTTTGCAAGCCTCAGCAGCGGCCTTGGGGTTGTTCTATTCGCGTCGTGCGCTGGACGACCCCGAGTA<br>CATCCGTGCGGCAGTTGTGCGTTTACTGGCCAAGATTCCGACAATGGTTGCAGCATTCCAACTTATGCGCAA<br>AGGAAACGACCCGGTACAACCGCGTGATGATCTGGATTATGCGGCTAACTTCTTGTACATGTTAAACGAACG<br>TGAGCCAGATCCGCTGGCCGCACGCATCTTCGACGTATGCCTTACGCTGCATGCGGAACATACTATCAACG<br>CCTCCACGTTCTCGGCAATGGTAACGGCGTCTACTTTAACTGACCCTTACGCAGTAGTAGCATCGGCTGTAG<br>GGACGTTAGCCGGCCCGTTACATGGAGGTGCAAACGAGGAGGTTTTGACAATGTTGGAGGAGATTGGCAG<br>TGTTGAGAACGTCCGTCCGTACTTAGACCATTGTATCGCGAAAAAGGCCAAGATCATGGGGTTCGGACACC<br>GCGTGTATAAAGTAAAGGATCCTCGTGCGACAATTTTACAAAATTTAGCAGAGCAGTTATTTGAGAAATTTGG<br>TCACGATCAGTATTATGAAATCGCACTGGAATTAGAGCGTGTAGTCGCTGAACGCCTTGGTCATAAAGGGAT<br>TTATCCGAACGTCGATTTTTATTCTGGATTGGTATACCGTAAATTAGGCATTCCATCGGATTTATTCACTCCG<br>GTATTTGCCATCGCACGCGTGGCAGGTTGGTTGGCCCACTGGAAAGAACAATTGTCCGAAAATCGCATCTT<br>CCGTCCAACCCAAATTTATACTGGATCACATAATATGACTTACGTGCCAATCGAAGAACGTGATTTGGCGAT<br>CGAATCTGAT
ancB k8R, y80F	ATGACGGCTGTCTCCGAGTTTCGTCCTGGACTTGAGGGTGTGCCCGCTACTCTGAGTTCTATTTCCTTCGTC<br>GACGGCCAGCGCGGGATTCTGGAATACCGTGGGATTAGTATTGAGCAATTAGCCCAACATTCGACATTTCTT<br>GAGACAGCGTATTTACTGATTTGGGGTAAACTTCCCACCCAAGAGGAATTAGCAGAATTCGAACATGAAATT<br>CGCTACCATCGCCGTATCAAGTTCCGTATTCGTGATATGATGAAGTGCTTCCCAGAGTCAGGTCACCCAATG<br>GATGCTTTGCAAGCCTCAGCAGCGGCCTTGGGGTTGTTCTATTCGCGTCGTGCGCTGGACGACCCCGAGTA<br>CATCCGTGCGGCAGTTGTGCGTTTACTGGCCAAGATTCCGACAATGGTTGCAGCATTCCAACTTATGCGCAA<br>AGGAAACGACCCGGTACAACCGCGTGATGATCTGGATTATGCGGCTAACTTCTTGTACATGTTAAACGAACG<br>TGAGCCAGATCCGCTGGCCGCACGCATCTTCGACGTATGCCTTACGCTGCATGCGGAACATACTATCAACG<br>CCTCCACGTTCTCGGCAATGGTAACGGCGTCTACTTTAACTGACCCTTACGCAGTAGTAGCATCGGCTGTAG<br>GGACGTTAGCCGGCCCGTTACATGGAGGTGCAAACGAGGAGGTTTTGACAATGTTGGAGGAGATTGGCAG<br>TGTTGAGAACGTCCGTCCGTACTTAGACCATTGTATCGCGAAAAAGGCCAAGATCATGGGGTTCGGACACC<br>GCGTGTATAAAGTAAAGGATCCTCGTGCGACAATTTTACAAAATTTAGCAGAGCAGTTATTTGAGAAATTTGG<br>TCACGATCAGTATTATGAAATCGCACTGGAATTAGAGCGTGTAGTCGCTGAACGCCTTGGTCATAAAGGGAT<br>TTATCCGAACGTCGATTTTTATTCTGGATTGGTATACCGTAAATTAGGCATTCCATCGGATTTATTCACTCCG<br>GTATTTGCCATCGCACGCGTGGCAGGTTGGTTGGCCCACTGGAAAGAACAATTGTCCGAAAATCGCATCTT<br>CCGTCCAACCCAAATTTATACTGGATCACATAATATGACTTACGTGCCAATCGAAGAACGTGATTTGGCGAT<br>CGAATCTGAT
AltaII ancB	ATGACGACTGTGTCAGAATTTAAACCGGGATTAGAGGGAGTCCCAGCAACGCAATCATCCATCTCATACGTC<br>GACGGCCAGAAGGGGATCCTGGAATATCGTGGTATTTCTATCGAGGAGCTTGCTAAACAGAGCACCTTTCTT<br>GAGACAGCTTACTTGCTTATCTGGGGAAAACTGCCGACTCAAGACGAATTGACCGATTTCGAACATGAGATC<br>CGTTACCATCGTCGCATCAAATACCGCATTCGCGATATGATGAAATGTTTCCCGGAGAGTGGTCACCCGATG<br>GATGCGTTACAAACAAGCGCAGCCGCGCTGGGACTGTTCTACTCGCGCCGTGCCCTGGATGACCCTGAATA<br>TATCCGCGCTGCGGTCGTGCGCCTGTTGGCTAAGATTCCGACTATGGTGGCTGCATTTCAACTTATGCGTAA<br>AGGGAATGACCCGGTCCAGCCACGTGACGATCTGGACTATGCCGCAAATTTCTTATACATGCTGAACGAGC<br>GTGAGCCCGATCCCTTAGCCGCCCGCATTTTCGATGTGTGCCTGACTTTACATGCAGAACACACTATTAATG<br>CCAGCACGTTCTCAGCTATGGTGACAGCATCCACTTTGACTGACCCATACGCTGTCGTGGCATCGGCAGTC<br>GGAACACTGGCAGGTCCCCTTCATGGCGGCGCTAATGAAGAGGTATTGACGATGTTAGAAGAAATCGGGTC<br>TGTTGAAAATGTACGTCCGTATCTGGATCACTGCATCGCTCGCAAGAGCAAGATCATGGGATTCGGCCACC<br>GTGTATATAAGGTGAAGGATCCGCGCGCTACGATTTTACAGAATTTAGCGGAGCAATTATTTGAGAAATTCG<br>GCCACGATCCATACTATGATATTGCCGTCGAGCTTGAGAAAGTGGTTGCGGAGCGCTTGGGTCACAAAGGG<br>ATCTATCCTAATGTAGACTTTTATAGTGGTTTGGTTTATCGTAAACTTGGTATTCCGACTGATCTGTTTACTCC<br>CATCTTTGCTATTGCACGCGTGGCGGGATGGTTGGCTCACTGGAAGGAACAGTTAAGTGAGAATCGTATCTT<br>TCGCCCGACGCAAATCTATACTGGATCTCACAACATGCCTTACATCCCCATCGAAGAGCGTGATTTGGCGAT<br>CGAATCTGAT
AltaII ancB q18L	ATGACGACTGTGTCAGAATTTAAACCGGGATTAGAGGGAGTCCCAGCAACGCTGTCATCCATCTCATACGTC<br>GACGGCCAGAAGGGGATCCTGGAATATCGTGGTATTTCTATCGAGGAGCTTGCTAAACAGAGCACCTTTCTT<br>GAGACAGCTTACTTGCTTATCTGGGGAAAACTGCCGACTCAAGACGAATTGACCGATTTCGAACATGAGATC<br>CGTTACCATCGTCGCATCAAATACCGCATTCGCGATATGATGAAATGTTTCCCGGAGAGTGGTCACCCGATG<br>GATGCGTTACAAACAAGCGCAGCCGCGCTGGGACTGTTCTACTCGCGCCGTGCCCTGGATGACCCTGAATA<br>TATCCGCGCTGCGGTCGTGCGCCTGTTGGCTAAGATTCCGACTATGGTGGCTGCATTTCAACTTATGCGTAA<br>AGGGAATGACCCGGTCCAGCCACGTGACGATCTGGACTATGCCGCAAATTTCTTATACATGCTGAACGAGC<br>GTGAGCCCGATCCCTTAGCCGCCCGCATTTTCGATGTGTGCCTGACTTTACATGCAGAACACACTATTAATG<br>CCAGCACGTTCTCAGCTATGGTGACAGCATCCACTTTGACTGACCCATACGCTGTCGTGGCATCGGCAGTC<br>GGAACACTGGCAGGTCCCCTTCATGGCGGCGCTAATGAAGAGGTATTGACGATGTTAGAAGAAATCGGGTC<br>TGTTGAAAATGTACGTCCGTATCTGGATCACTGCATCGCTCGCAAGAGCAAGATCATGGGATTCGGCCACC<br>GTGTATATAAGGTGAAGGATCCGCGCGCTACGATTTTACAGAATTTAGCGGAGCAATTATTTGAGAAATTCG<br>GCCACGATCCATACTATGATATTGCCGTCGAGCTTGAGAAAGTGGTTGCGGAGCGCTTGGGTCACAAAGGG<br>ATCTATCCTAATGTAGACTTTTATAGTGGTTTGGTTTATCGTAAACTTGGTATTCCGACTGATCTGTTTACTCC<br>CATCTTTGCTATTGCACGCGTGGCGGGATGGTTGGCTCACTGGAAGGAACAGTTAAGTGAGAATCGTATCTT<br>TCGCCCGACGCAAATCTATACTGGATCTCACAACATGCCTTACATCCCCATCGAAGAGCGTGATTTGGCGAT<br>CGAATCTGAT
ancC	ATGGCGGTCTCTGAGTACAAGCCGGGTTTAGAGGGTGTACCTGCGACGCAGTCATCTATCTCGTTTGTCGA<br>TGGCCAACGTGGTATCCTGGAATACCGCGGCATTAGCATCGAAGAGCTGGCAAAACACTCGACATTTCTGG<br>AAACTGCTTACTTACTGATTTGGGGTAAATTACCCACACAAGAAGAACTGGCGGAGTTTGAGCATGAGATCC<br>GCTACCATCGTCGCATCAAGTATCGTATCCGCGACATGATGAAGTGTTTCCCCGAGTCTGGTCATCCAATGG<br>ATGCATTACAGGCTTGCGCCGCGGCTCTGGGTTTATTCTATTCGCGTCGTGCTCTGGACGATCCTGAGTACA

	TTCGCGCGGCTGTTGTGCGTCTTTTAGCTAAGATTCCGACTATGGTTGCAGCCTTCCAACTGATGCGCAAAG<br>GAAACGACCCTGTTCAACCTCGTGACGACTTGGACTATGCAGCAAATTTTTTGTATATGCTTAATGAGCGCG<br>AGCCAGATCCCTTGGCGGCACGCATTTTCGATGTTTGCTTAACGTTACACGCTGAGCATACAATCAACGCTT<br>CTACGTTTAGTGCGATGGTCACAGCCTCAACATTGACTGATCCTTACGCCGTCGTCGCCTCAGCGGTTGGC<br>ACCCTTGCGGGCCCGTTACATGGGGGCGCCAACGAAGAAGTACTTACTATGCTGGAAGAGATCGGGTCTGT<br>GGAAAATGTGCGCCCCTATTTGGAGCACTGCATTGCCCGCAAGGCAAAGATCATGGGTTTTGGTCATCGTG<br>TTTACAAAGTGAAAGACCCTCGTGCCACCATCTTGCAGAACCTGGCTGAACAATTATTTGAGAAGTTCGGCC<br>ACGACCAGTACTACGATATTGCATTAGAACTTGAGCGTGTGGTTGCAGAACGTCTGGGTCACAAAGGTATCT<br>ACCCGAACGTCGATTTCTACTCTGGCCTGGTTTACCGTAAACTTGGCATCCCTACCGATTTGTTTACCCCCG<br>TTTTCGCTATTGCACGCGTCGCAGGCTGGCTGGCACACTGGAAGGAACAGCTGTCTGAGAATCGTATTTTC<br>CGTCCGACACAGATCTATACAGGTTCCCATAACATGCCGTATGTGCCCATTGAGGAACGT
ancC q18L	ATGGCGGTCTCTGAGTACAAGCCGGGTTTAGAGGGTGTACCTGCGACGCTGTCATCTATCTCGTTTGTCGA<br>TGGCCAACGTGGTATCCTGGAATACCGCGGCATTAGCATCGAAGAGCTGGCAAAACACTCGACATTTCTGG<br>AAACTGCTTACTTACTGATTTGGGGTAAATTACCCACACAAGAAGAACTGGCGGAGTTTGAGCATGAGATCC<br>GCTACCATCGTCGCATCAAGTATCGTATCCGCGACATGATGAAGTGTTTCCCCGAGTCTGGTCATCCAATGG<br>ATGCATTACAGGCTTGCGCCGCGGCTCTGGGTTTATTCTATTCGCGTCGTGCTCTGGACGATCCTGAGTACA<br>TTCGCGCGGCTGTTGTGCGTCTTTTAGCTAAGATTCCGACTATGGTTGCAGCCTTCCAACTGATGCGCAAAG<br>GAAACGACCCTGTTCAACCTCGTGACGACTTGGACTATGCAGCAAATTTTTTGTATATGCTTAATGAGCGCG<br>AGCCAGATCCCTTGGCGGCACGCATTTTCGATGTTTGCTTAACGTTACACGCTGAGCATACAATCAACGCTT<br>CTACGTTTAGTGCGATGGTCACAGCCTCAACATTGACTGATCCTTACGCCGTCGTCGCCTCAGCGGTTGGC<br>ACCCTTGCGGGCCCGTTACATGGGGGCGCCAACGAAGAAGTACTTACTATGCTGGAAGAGATCGGGTCTGT<br>GGAAAATGTGCGCCCCTATTTGGAGCACTGCATTGCCCGCAAGGCAAAGATCATGGGTTTTGGTCATCGTG<br>TTTACAAAGTGAAAGACCCTCGTGCCACCATCTTGCAGAACCTGGCTGAACAATTATTTGAGAAGTTCGGCC<br>ACGACCAGTACTACGATATTGCATTAGAACTTGAGCGTGTGGTTGCAGAACGTCTGGGTCACAAAGGTATCT<br>ACCCGAACGTCGATTTCTACTCTGGCCTGGTTTACCGTAAACTTGGCATCCCTACCGATTTGTTTACCCCCG<br>TTTTCGCTATTGCACGCGTCGCAGGCTGGCTGGCACACTGGAAGGAACAGCTGTCTGAGAATCGTATTTTC<br>CGTCCGACACAGATCTATACAGGTTCCCATAACATGCCGTATGTGCCCATTGAGGAACGT
AltaII ancC	ATGACAGTGTGTGAATATAAGCCCGGACTGGAGGGGGTGCCTGCAACTCAATCATCAATCAGTTACGTTGA<br>CGGTCAGAAGGGCATCTTGGAGTACCGTGGTATTCAGATTGAGGAATTGGCACAGCATTCCACCTTTTTGGA<br>AACAGCATACCTGTTAATCTGGGGAACTTTACCGACGCAGGAGGAGCTTACGGACTTTGAGCATGAAATCC<br>GCTATCACCGCCGTATCAAATACCGCATTCGTGATATGATGAAGTGCTTTCCAGAATCTGGACATCCCATGG<br>ACGCGCTGCAGACGTGCGCTGCTGCCCTTGGTCTTTTCTACTCGCGTCGCGCTCTGGACGACCCAGAGTAC<br>ATCCGCGCGGCTGTCGTCCGCTTGTTAGCTAAAATCCCAACAATGGTGGCCGCGTTCCAGTTGATGCGTAA<br>AGGTAATGACCCAGTTCAACCTCGCGATGATTTGGACTATGCGGCGAATTTCCTTTATATGTTAAATGAACGC<br>GAGCCCGATCCACTTGCGGCGCGTATCTTCGACGTGTGTTTAACCTTACATGCTGAACATACGATTAACGCC<br>AGCACATTTTCTGCCATGGTAACAGCCTCGACATTGACGGACCCTTATGCGGTCGTGGCCTCAGCTGTAGG<br>AACTCTTGCCGGTCCTCTTCACGGTGGGGCTAATGAGGAGGTTATCACTATGTTGGAGGAGATTGGATCCG<br>TCGAGAACGTGCGTCCGTACCTGGATAATTGTCTGGCCCGTAAAGCAAAGATTATGGGATTCGGCCACCGC<br>GTCTACAAGGTCAAGGATCCTCGCGCGACCATCCTTCAGAATCTGGCCGAACAATTATTTGAGAAATTTGGC<br>CATGATCCGTATTACGACATTGCCATCGAGTTGGAGAAAGTGGTTTCCGAGCGTCTTGGTCACAAGGGTATC<br>TATCCGAATGTCGACTTCTACTCGGGGTTGGTGTACCGCAAACTGGGCATCCCAAGCGACCTGTTTACACC<br>CATCTTCGCCATTGCACGTGTTGCTGGGTGGCTTGCTCATTGGAAGGAACAGTTATCCGAGAACCGTATCTT<br>TCGCCCCACACAGATCTACACAGGGAGCCACAATATGCCGTATATCCCCATTGAAGAGCGTGATTTGGCGA<br>TCGAATCTGAT
ancD	ATGGCGGTTGGCGAGTATAAACCGGGCCTGGAAGGTGTACCAGCGACGCAATCTAATATTAGTTTCGTCGA<br>TGGGCAACGTGGTATCTTGGAATATCGTGGTATTCCTATTGAGGAATTAGCAAAGCACAGTACTTTTTTAGAA<br>ACTGCCTATCTTCTGATCTTCGGTAAACTTCCGACACAGGATGAATTAAAGGAGTTCGAGCATGAGATTCGT<br>CACCATCGCCGTATCAAATACCGTATTCGCGACATGATTAAGTGCTTCCCTGAGTCCGGCCACCCTATGGAC<br>GCCCTGCAAACATGTGTCGCTGCTCTTGGACTGTTCTACCCGTTACGCGAATTAGATGATCCTGACTATATT<br>CATGGGGCCACGGTACGCCTGCTGGCAAAGATCCCCACCATGGTGGCTGCATTCCACATGATGCGTCAGG<br>GCAATGACCCCGTTCAGCCCCGCGACGACCTTGATTACGCGGCAAATTTCTTGTATATGTTGAATGAGAAAG<br>AACCGGATCCATTAGCCGCACGTATCTTCGATGTCTGCTTAATCCTTCACGCAGAACACACGGTCAATGCTT<br>CTACCTTCTCCGCCTTGGTTACTGCTAGTACTTTAACAGATCCATATACGGTTATCGCTTCCGCTGTCGGCAC<br>CCTGTCGGGGCCTCTTCACGGAGGAGCAAATGAACAGGTAATGACCATGTTAGAGGAGATCGGTAGCGTC<br>GAAAATGTCCGTCCGTACCTTGAGCGCAAGCTTGCGCGTAAGGAAAAAATCATGGGATTTGGACATCGTGTT<br>TATAAAGTCAAAGATCCGCGCGCAACCATCCTTCAAAACCTTGCCGAGGAGCTTTTTGACAAATTCGGACAC<br>GATCCCTACTATGACATCGCGCTTGAGCTGGAGCGTGTGGCCGCTGAACGCCTGGGCCACAAGGGTATCTA<br>CCCTAATGTTGACTTTTACTCAGGCCTTGTATATAAAAAACTGGGCATTCCAACCGACCTGTTTACTCCAGTT<br>TTCGCTATTGCTCGTGTCGCGGGGTGGCTGGCACACTGGAAAGAGCAATTGAGTGACAACCGCATCTTTCG<br>CCCCACCCAGGTCTACACTGGAGAGCATAACGTACCTTACGTTCCTATTGCAGAACGCGATTTGGCGATCG<br>AATCTGAT
ancE	ATGGCAGACGGCGAATATAAACCCGGCTTAGAGGGTGTACCGGCCACTCGTAGCAATATCAGCTTCGTAGA<br>TGGTCAAGCAGGGATCCTTGAATATCGTGGTATTCCAATTGAGGAACTTGCTGAACATAGTACGTTCTTGGA<br>GACGGCTTACTTACTGATTTTCGGCAAGTTACCCACACAGGATGAGTTAGATGAGTTCGAGCACGAGATTCG<br>TAGCCACCGTCGTGTCAAATACCGCATCCGTGATATGATTAAGTGTTTTCCAGAATCCGGCCATCCGATGGA<br>CGCGTTGCAAACATGCGTGGCTGCATTGGGCCTGTTTTACCCATTACGTGAGATGGACGATGACGACTATG<br>TGCACGGCGCTACAGTACGCTTGCTTGCCAAAATCCCCACTATGGTGGCAATGTTCCATCAAATGCGCCAA<br>GGGAATGATCCTATCCCGCCCCGTGATGATTTGGATCATGCGGCTAATTTTTTGTATATGTTAACAGGAAAA<br>GAACCTGATCCTCTGGCAGCCCGCATTTTTGATGTTTGTTTGATCTTGCATGCCGAGCACACAGTGAATGCC<br>AGCACATTCTCGGCCTTGGTTACAGCTTCCACCTTAGCTGACCCCTACACAGTGATCGCTAGCGCCGTTGG<br>AACACTTTCAGGCCCTCTGCATGGTGGTGCTAACGAACAAGTGATGCAAATGTTAAAAGAGATCGGTTCTGT<br>AGAGAACGTCCGTCCGTATATTGAGGAGAAATTGGCCCGCAAAGAGAAGATCATGGGGATGGGCCACCGT<br>GTATATAAAGTGAAGGACCCACGCGCCACCATCTTACAGAACTTAGCGGAAGAGTTGTTCGACCGTTTCGG<br>ACATGACCCATATTATGACATCGCTCTGGAAGTAGAGCGCGTGTGTGAGGAGCTGCTGGGTCACAAAGGCA<br>TCTATCCTAATGTTGACTTCTACTCGGGTTTAGTCTATTCAAAACTTGGGATTCCCACGGACTTATTTACCCCT<br>GTATTCGCAATCGCCCGCGTGGCCGGATGGTTGGCACACTGGCGCGAACAATTGTCAGACAACCGTATTTT<br>TCGCCCTACCCAAGTATATACCGGGGAACGCAATGTCCCGTATACACCGTTATCAGAACGCGATTTGGCGAT<br>CGAATCTGAT

CS *Methylophaga sulfodovorans*<br><br>NCBI Reference Sequence: WP_091715369.1	ATGAGCGACTTTCTTCCTGGGCTTGAAGGGGTTCCAGCTACCAAAAGCGCAATTTCTTTCATTGACGGGGAG<br>AAAGGCATTCTTAGCTATCGCGGTTATCCTCTGGAGACGCTTGCTGAAAATAGCACTTTCGAGGAAACGACG<br>CTTCTGCTTTTAGATGGCGAGTTGCCGACGAAGAAGGCGCTTAATGACTTTAGCCAGCAACTGAAGGACAAT<br>TATCGCATCAAGTATCACATTCGCCAAATGATGCGTCATTTTCCTCATACAGGACATCCGATGGATATGCTTC<br>AGACAGCCGTTTCCTCTTTAGGCATGTTTTATCCCGGCACTGAATGCCTGACTGACGCCAACTCCTGCGAAG<br>ACCTGGACTATGTTCGCAATATGACAGTGAATATTATTGCACAGATGGCCCCATTAGTGGCGATGTGGGAAC<br>ATATCCGTAATGGATGGGACCCTGTTAACCCAAAGCATGACCTTAGCGTCGCCGAGAACCTGCTTTACATGT<br>TCAATGGGGAAGAGCCGGATCCTCTGATGGCGAAAATCATGGATGTGTGCCTGATCCTGCATGCCGAGCAT<br>ACACTGAATGCTTCTACCTTTGCCGCGTTAGTAGCTGGGTCAACACTGGCGACCCCATACTCCGTTATCAGT<br>GCGGCAATCGGGACATTGTCGGGTCCATTGCATGGTGGAGCGAATCAGCGTGTCGTTGGCATGCTGCAGG<br>AGATTGGGAGCCCGAAGAACGTCGAAAGTTGGGTAGACGAAAAATTAAAAAACAAGGAAGTTATTTGGGGA<br>ATGGGTCACCGCGAATATAAGGTCAAGGACCCGCGCGCGACTATTTTGCATAAACTGGTAGAGCAGTTGGT<br>AGCCGAACGTGGGGGTCACCTTGACGATATGTTTGACACAGCCTTAAAACTTGAGGAGGTTTGCGCTGATC<br>GTCTTGGGCACAAAGGCGTTTATCCTAATGTAGACTTTTATTCTGGCATCTTGTATTCGGAAATGGGTATCCC<br>CGAGGACGAGTTTACGGCCTTGTTCGCTGTGGCTCGCAGCGCAGGATGGCTGGCTCATTGGCGTGAACAG<br>ATTTCAGATAATCGTATCTATCGTCCTACCCAAATCTATGTCGGCTCCGATATGCGCGATTACACACCAATCG<br>AAGAGCGC
CS *Gloeobacter violaceus*<br><br>NCBI Reference Sequence: WP_011143005.1	ATGTCAGGGGAGTATGTGCCTGGATTAGAAGGCGTACCAGCAACACGTTCAAATATCTCGTTTGTGGACGG<br>AAAAGCAGGTGTGTTAGAGTATCGCGGTATTCCGATCGATCAGTTGGCCGAGTCATCTACATTTTTAGAAAC<br>AGCTTTTTTGCTGATTTTCGATCATTTGCCTACGAAGGACGAATTATTGAGCTTCGAAGTTGAGATTTTAGGC<br>CATCGTCGTGTAAAATATCGCATTCGCGACATGATCAAAAGCTTCCCCGAGAGTGGCCGTCCCATGGTCGC<br>CCTTCAGTCTTGCATCGCGGCGCTTGGCTTATTTTACCCCTTGCAGAACGAATCGAAAGAAAAATACGCCTA<br>CGATAGTACTATCCGTTTATTAGCTAAAATGCCCACCATGGTAGCCACCTTTCATCAAATGCGCCTGGGGAA<br>CGATCCCATCCCACCACGCGATGATTTGGGGCATGCCGCAAACTTCTTGTATATGTTGACGGGAAAAGAAC<br>CGGACCCGCGTGCAGCTCGTATCTTTGACGTTTGCCTGATGTTGCATGCAGAACACACGGTCAATGCTTCTA<br>CCTTCAGCGCCCTTGTTACGGCATCAACTTTGGCAGACCCATACACTGTAATCACCAGTGCGGCAGGAACAT<br>TGTCCGGCCCTCTGCACGGAGGTGCGAACGAGGAAGTCATTCGCATGTTAAAGGAAATCGGCACCATCGAA<br>CGTGTACGCCCATACTTAGAAAATCGTTTAGCTCGTAAAGAAAAGATCATGGGCGTCGGCCACCGCGTATAT<br>AAGGTGAAAGACCCACGCGCAACGATTCTTCAAAACTTAGCTCAAGAGCTTTTTGATCGCTTCGGCCACAGC<br>CGTTTATACGACATTGCCGTGGAAGTGGAGCGCGTTTGCGACGAGCTTTTGGGTCAAAAAGGAATCTATCC<br>GAATGTAGATTTCTATTCAGGGTTGGTCTATGAGAAAATGGGGATTCCGGCTGATATGTTTACACCGGTCTTT<br>GCGATTTCCCGCGTCGCCGGATGGCTGGCACATTGGCATGAACAGTTGGCGGACAATCGTATTTTCCGCCC<br>AACGCAAGTTTATACTGGCAGCCATAACGTGGAGTTCACGCCCCTTTCGTTGCGCAGCTACGCA
CS *Pseudanabaena biceps*<br><br>NCBI Reference Sequence: WP_175355653.1	ATGGCCATCGGCGAGTATAAACCAGGATTAGAAGGCGTGCCCGCGACCCAGAGCAACATTTCATACGTCGA<br>CGGAAAAGCGGGGCTTCTGGAGTATCGTGGGATCCGTATCGAAGAATTGTGCGTACACAGTAGTTTCTTGG<br>AGACATCATATCTGTTAATCTTTGGTGAGTTACCTACGTCGGCGAAGTTAAAGGAGTTTGAAGTTGATATTAC<br>CCACCGTCGTCGCATCAAATATCGTATCCGTGATATGATTAAATCGTTTCCTGATAACGCACACCCCATGGAT<br>GCCTTGCAAACCAGTGTGGCGGCACTGGGCATGTTTTATCCGTTGGGAGATTTCCACGACGCGGATTATAT<br>CTATCAGGCGACCGTGCGTCTGCTTGCCAAAGTGCCCACTATGGTTGCAGCCTTCCATATGATGCGTCAAG<br>GCAATGATCCAGTTATGCCACGCGACGATCTGGACTATGCGTCGAACTTCCTTTACATGCTGAATGAGAAAG<br>TGCCGGACCCTTTAGCCGCGCGTATCTTTGACGTTTGTCTGACTTTACACGCGGAACACACTGTTAATGCAA<br>GCACGTTTGCGGCGTTGGTCACGGCTTCAACGTTAACGGATCCCTACGCGGTTATTACATCGGCTATCGGA<br>ACGTTAGCCGGCCCACTGCACGGGGGCGCCAACGAACAAGTGATGATGATGTTGGAGGAAATCGGTTCCG<br>TTGATAATGTTACCGCGTATTTAGAACGTAAGATCGAGCGCAAGGAGAAGCTTATGGGCTTTGGCCACCGTA<br>TTTACAAAGTAAAGGATCCGCGTGCTATTGTATTGCAAGAGTTAGTCCATAAAATGTTTGACCAGTTCGGACA<br>TGACCATTACTACGATATCGCCTTGGAGTTAGAGAAACAGGCATTTGAGAAACTTTCGTCAAAGGGAATCCA<br>CCCGAACGTAGATTTTTACTCTGGGTTAGTCTACAAGAAGCTTGGAATCCCTAGCAACTTATTCACGACAATC<br>TTCGCTATCGCCCGTGTGCCAGGTTGGCTTGCTCATTGGAAAGAACAATTGAGTGACAATCGTTTATTCCGC<br>CCGACTCAGGTCTACACGGGGCTTCATGATGTTACGTATTTACCTATTGAACATCGT
CS *Cyanothece sp. SIO1E1*<br><br>GenBank: NET35916.1	ATGACATTCTGTGAATATAAACCCGGGCT?GAAGGAATTCCGGCGACCCAAAGTTCAGTAAGTTATGTTGAT<br>GGTCAGCGTGGAATCCTGGAGTATCGCGGCATCCAAATCGAAGCACTTGCCGCTAAGTCAAATTTCCTTGA<br>GACAGCCTACCTTTTGATCTGGGGTGGTTTACCTACTCATGAGGAGTTGGCGTCATTTGAATCGGAAATTCG<br>CTACCACCGTCGTTTGAAGTACCGTATCCGCGACATGATGAAGTGCTTTCCGGAAAGCGGTCACCCGATGG<br>ATAGCCTGCAGGCGTGTGCAGCCGCCCTGGGTCTGTTCTATTCGCGTCGCGCACTTGATAACCCTGTTTAT<br>ATTCGCGCTGCGGTAGTTCGTCTGCTTGCCAAAATCCCTACTATGGTAGCTGCTTTTCAGATGATGCGCAAA<br>GGAAATGACCCCATCCAACCGCGCGACGATCTGAGCTACTCTGCCAACTTTCTGTATATGCTTAATGAACGT<br>GAACCGGATCCGTTAGCTGCTCACATCTTCGATGTGTGTCTTACGCTTCATGCCGAGCATACTATCAACGCG<br>TCAACCTTTTCCGCAATGGTGACCGCCAGTACACTTACAGATCCCTACGCGGTTGTCGCGTCAGCCGTGGG<br>TACATTAGCAGGACCCCTTCATGGAGGCGCGAATGAAGAGGTGCTTTCTATGCTGGAGGAAATTGGAAGCG<br>TGGGTAATGTGCGCCCGTACTTGGAAGATTGTCTGCAGCGCAAAGCGCGCATTATGGGATTCGGTCACCGC<br>GTGTACAAGGTCAAAGATCCACGCGCCATTATTCTGCAAGACCTGGCAGAACAGTTGTTTGAAAAGCTTGGT<br>GGCGATCGTTACTACGACATTGCAGTAGAGCTTGAGCGCCAGGTCTCTGAAAAATTGGGGCACAAAGGCAT<br>CTATCCAAACGTCGACTTTTATTCGGGATTGGTCTATCGTAAGTTGGGTATTCCCCGCGATATGTTCACGCC<br>GGTCTTCGCTATTGCCCGTGTTGCTGGGTGGTTGGCGCATTGGAAGGAGCAACTTGCTGAGAACCGCATCT<br>TTCGTCCGACTCAGATCTACACTGGACCTCGCCACATTCCTTATGTAGCTATTGCTGATCGTCATCCACCCC<br>AGGAGGAGGTCATTCTTACTAATTTACTTGCAGAT
CS *Planktothrix mougeotii*<br><br>NCBI Reference Sequence: WP_193869840.1	ATGACAGCCGTCTCCGAATTCAAACCTGGCCTTGAGGGGGTACCAGCTACACTGTCGTCAATTTCATACGTG<br>GATGGGCAAAAGGGTATCCTTGAATATCGTGGCATCTCCATCCAACAGTTGGCTAATTACTCAACCTTTTTG<br>GAGACGAGTTATTTATTAATCTGGGGCAAATTACCGACTAAAGACGAACTGGAAGCATTTGAGCATGAAATT<br>CGCTATCACCGTCGCATTAAGTATCGTATTCGTGATATGATGAAATGTTTTCCGGAAACTGGACACCCTATG<br>GATGCATTACAGACGTCCGCTGCAGCCTTGGGCCTGTTTTATAGTCGCCGCGCTTTAGACAACCCCCAGTA<br>CATTCGTGAAGCAGTCGTTCGTTTGTTGGCAAAAATCCCAACTATGGTCGCGGCCTTCCAATTAATGCGCAA<br>AGGAAACGATCCAGTGCAACCGCGCGATGACCTTGATTACTCAGCTAATTTCTTATATATGTTGTCTGAGCG<br>TGAGCCAGACCCCCTTGCCGCGTGGGTTTTCGACGTTTGCCTTACGCTTCACGCGGAACACACAATCAATG<br>CAAGTACATTCAGCGCAATGGTCACAGCTAGTACTTTGACGGATCCTTATGCAGTCATTGCTTCAGCAGTAG<br>GCACATTAGCCGGTCCGTTGCACGGTGGGGCTAACGAGGAGGTGTTAGTAATGCTGGAGGAAATTGGGGC<br>AGTTGAGAACGTTGGTCCTTATGTGGATAACTTGATTGCCAAGAAAAGTAAAATCATGGGATTTGGCCATCG<br>CGTTTATAAAGTTAAAGACCCTCGTGCTACAATTTTGCAACAGTTAGCTGAGAAGCTGTTCGAGAAGTTTGG<br>GCATGACCAGTACTATGAAATTGCCTTGGAACTTGAAAAAGTCGTAGCGGACCGTTTAGGTGGGAAGGGGA<br>TCTATCCGAATGTCGACTTCTATAGTGGCCTGGTGTACCGCAAGTTGGGAATCCCCGCTGACTTATTTACTC<br>CGATCTTCGCTATCGCCCGTGTGAGTGGGTGGCTGGCTCATTGGAAAGAGCAATTAACAAAAAATCGCATCT

	TTCGCCCAACTCAAATCTATACGGGCAGTCACAATGAGACTTATGTGCCTATCCATGAGCGTAATTGTGCAA<br>TTAACCTTGAAGAATCAACGTTG
Δ2-6 CS Planktothrix mougeotii	ATGTTCAAACCTGGCCTTGAGGGGGTACCAGCTACACTGTCGTCAATTTCATACGTGGATGGGCAAAAGGG<br>TATCCTTGAATATCGTGGCATCTCCATCCAACAGTTGGCTAATTACTCAACCTTTTTGGAGACGAGTTATTTAT<br>TAATCTGGGGCAAATTACCGACTAAAGACGAACTGGAAGCATTTGAGCATGAAATTCGCTATCACCGTCGCA<br>TTAAGTATCGTATTCGTGATATGATGAAATGTTTTCCGGAAACTGGACACCCTATGGATGCATTACAGACGTC<br>CGCTGCAGCCTTGGGCCTGTTTTATAGTCGCCGCGCTTTAGACAACCCCCAGTACATTCGTGAAGCAGTCG<br>TTCGTTTGTTGGCAAAAATCCCAACTATGGTCGCGGCCTTCCAATTAATGCGCAAAGGAAACGATCCAGTGC<br>AACCGCGCGATGACCTTGATTACTCAGCTAATTTCTTATATATGTTGTCTGAGCGTGAGCCAGACCCCCTTG<br>CCGCGTGGGTTTTCGACGTTTGCCTTACGCTTCACGCGGAACACACAATCAATGCAAGTACATTCAGCGCAA<br>TGGTCACAGCTAGTACTTTGACGGATCCTTATGCAGTCATTGCTTCAGCAGTAGGCACATTAGCCGGTCCGT<br>TGCACGGTGGGGCTAACGAGGAGGTGTTAGTAATGCTGGAGGAAATTGGGGCAGTTGAGAACGTTGGTCC<br>TTATGTGGATAACTTGATTGCCAAGAAAAGTAAAATCATGGGATTTGGCCATCGCGTTTATAAAGTTAAAGAC<br>CCTCGTGCTACAATTTTGCAACAGTTAGCTGAGAAGCTGTTCGAGAAGTTTGGGCATGACCAGTACTATGAA<br>ATTGCCTTGGAACTTGAAAAAGTCGTAGCGGACCGTTTAGGTGGGAAGGGGATCTATCCGAATGTCGACTT<br>CTATAGTGGCCTGGTGTACCGCAAGTTGGGAATCCCCGCTGACTTATTTACTCCGATCTTCGCTATCGCCCG<br>TGTGAGTGGGTGGCTGGCTCATTGGAAAGAGCAATTAACAAAAAATCGCATCTTTCGCCCAACTCAAATCTA<br>TACGGGCAGTCACAATGAGACTTATGTGCCTATCCATGAGCGTAATTGTGCAATTAACCTTGAAGAATCAAC<br>GTTG
CS *Cyanobium sp. PCC7001*<br><br>NCBI Reference Sequence: WP_006910478<br>.1	ATGGCCGGGAGCTTGAGTGATAGTGTACCTGGATCGACTGGAGGGGCAACGGCTGCCCCACCATTTCGTC<br>CCGGGTTGGAAGGTGTACCAGCAACTCAGAGCGCGATCTGTGACATTGATGGACAAAAGGGTCGTTTAACA<br>TACCGCGGGTACGACGCGGGGGAGCTGGCCGCTCATAGCACGTTCTTGGAAACAACCTACTTATTAATTTG<br>GGGAGAGCTTCCCACCGCCGAAGGTTTGCGTCAGTTTGAACACGAGGTTCAGATGCATCGCCGTGTTTCCT<br>TCCGTATTCGTGACATGATGAAATGCTTCCCCGCTACAGGCCATCCCATGGATGCATTGCAGTCGTCCGCC<br>GCCAGCTTGGGTCTGTTTTATTCCCGCCGTGCGCTGGACAATCCCGAGTATATTGCAGAAGCGGTGGTACG<br>CTTAATTGCCAAAATTCCCACTATGGTAGCAGCCTTCCAACTGATTCGCAAGGGCCAGGATCCGATCCAACC<br>GCGCGACGATCTTCCATTCGCGTCGAACTTTCTTTACATGCTGACTGAGCAGGAACCTGATCCTCTGGCGG<br>CTCGCATCTTCGATGCATGTCTGATCTTACATGCTGAGCATTCTCTGAACGCAAGCACGTTCTCGGCGCGCG<br>TCACTGCTTCTACTTTGACTGACCCCTACGCCGTCGTGGCGAGCGCAGTTGGCACGTTGGCTGGCCCACTG<br>CACGGTGGCGCAAACGAGGATGTCCTGGCCATGTTGGAGGCTATCGGGAGTGCAGACCAGGTGGAACCGT<br>GGTTGGACCGTGCAATCGCCCAGAAACAAAAGATTATGGGTTTTGGTCATCGCGAGTACAAGGTGAAAGAC<br>CCTCGCGCCGTGATTTTACAGGGCTTAGCGGAACAGCTTTTCCACCGTTTCGGGCACGACCCCTTGTATGA<br>CTTAGCGCGTAAACTGGAAGAAGCTGCGGCAGAACGCTTAGGCCCGAAAGGTATCTACCCGAACGTAGATT<br>TTTATTCCGGCTTGGTCTACCGTAAGTTAGGGATCCCGCGCGATCTTTTTACACCTATTTTTGCCATCGCCCG<br>CACAGCAGGTTGGCTTGCGCACTGGAAGGAGCAATTAGGTGCAAACCGCATCTTCCGTCCATCACAGATCT<br>ACACAGGACCTGTACCCCGCGACTGGGTCCCCCTTGAAGCCCGT

Supplementary table 3 List of homology cassettes used for transformation of *S.elongatus* PCC7942

CS variant	DNA sequence full homology cassette
Wildtype SeCS	GGCAGCAAACGCCGCAATCTCCAGATTCAAGCCACAGTACGGGGTGGCCATGGTCGCGATGGTGATTTTGAAGT<br>GTTTGCCACCTCCCTCGATCGCCTTGCGACAGCACTCGAAGGCCGCGATTCCCTGACGACAAATCCTGACTTCC<br>GCCAAGCCCAGCGCCAACTACCCCAACCCAATCAAGGTGTCCTCTACCTTGACTGGCAGGCCAGTGCCCTACC<br>GCTGACGCAACGCTGGCCTGTCTTGCAGCTGATTGCCCTGCCCTTGCAACCCCTGTTGGAGCACGTGCAGGCG<br>ATCGCGATCGCCAGTCCTACCCCTCCCGAAGACAGCACTGACCTGCAGCGACTCTCAATCGAACTGCTGTTGCG<br>GAGCTAGGCGATGGAACTCTATCTGATCCGCCATGGCATCGCCGCCGAACGGGGCACCTACGCGGATGATGAC<br>CGGCGCCCCCTGACAGCCACGGGAGAACGCCGCAGTCAACGAGTTGCCCAGCGACTGCTGTCTCTCGGTCTGC<br>ATTTCGATGTTCTGCAAACCAGTCCCCTAGTCCGCGCCCACCAAACCGCCGTCATCCTTCAACAAGAGGGGCTG<br>GCTTCCCAAATCGCGATCGCCCCAGAATTAGCGCCCGAAGGCAGTCTGACCGCTTGGCTCCGGCGCTTACCGC<br>CCGCCATCTCGGCTGATCAGCGCTGGGCGATCGTGGGCCATGAACCCGATCTAGGGGTCTGGGCGGAACAGCT<br>GGTCTGGGGATCTGCCCAGGACAAGCTCGTTCTCAAGAAAGCCGGTCTGATCGGTCTGCAATTCCCAGGCGATC<br>GCCCTGCGATCGGAGCCGGATCCCTCTTCTGGCTAACCCCCCCGCGCCTTCTCCTTTAGGGGCACCTGATCAAA<br>TTGGCGAAAGACGCGCGGCAGACCCACCATCAGGTTTCGTGCCTGTTGCGACGAAGATGCACCAGCCAGACTG<br>ATAGGGTATTCGGAAGAAAGACTGCATGGGGAAGCCCAGGATTGCTATGACTGCCGTCAGCGAGTTTCGGCCTG<br>GCCTAGAAGGCGTGCCCGCCACACTCTCGAGCATTAGCTTTGTCGATGGCCAGCGCGGCGTCCTAGAGTATCG<br>CGGCATCAGCATCGAGCAACTGGCGCAACAGAGCAGTTTTCTGGAAACCGCCTACCTGTTGATTTGGGGCCATC<br>TACCAACTCAGCAGGAATTGACCGAGTTCGAGCACGAAATTCGCTACCACCGCCGCATCAAGTTCCGCATCCGG<br>GACATGATGAAATGCTTCCCCGATAGCGGCCATCCTATGGATGCCCTGCAGGCGAGCGCCGCAGCCCTCGGGT<br>TGTTCTATTCGCGGCGCGCCTTGGATGATCCCGAATACATTCGGGCGGCCGTTGTGCGTTTGCTAGCCAAAATT<br>CCGACGATGGTGGCTGCCTTCCAGCTGATCCGCAAGGGTAACGACCCAATTCAGCCCCGCGATGAACTGGACT<br>ACGCCGCCAACTTTCTCTACATGCTGACGGAGCGCGAGCCCGATCCAGTCGCAGCTCGGATTTTTGATATTTGC<br>CTCACCCTGCACGCCGAACATACGATCAACGCCTCGACCTTCTCGGCGATGGTCACAGCTTCGACCCTGACCGA<br>TCCCTACGCTGTCGTTGCTTCTGCCGTTGGCACCTTGGCTGGCCCCCTCCATGGCGGCGCCAATGAAGAAGTG<br>CTGGACATGCTGGAGGCGATCGGTTCCGTCGAGAATGTTGAGCCCTACCTCGACCACTGCATTGCCACCAAAAC<br>GCGCATTATGGGCTTTGGGCACCGTGTCTACAAAGTCAAGGATCCGCGGGCAGTCATTCTGCAAAATCTGGCCG<br>AGCAACTGTTCGATATCTTCGGCCATGATCCCTACTACGAAATCGCGGTCGCAGTCGAAAAGGCAGCAGCCGAG<br>CGACTCAGCCACAAGGGCATTTACCCCAACGTCGATTTCTACTCCGGCTTGGTCTATCGCAAGCTCGGTATTCCT<br>AGCGATCTATTCACACCGGTGTTTGCGATCGCGCGGGTTGCGGGCTGGCTCGCCCACTGGAAAGAGCAGCTGA<br>ACGAAAATCGGATCTTCCGCCCCACTCAGATCTACACGGGCAGCCACAACCTCGACTACACCCCGATCGCCGAT<br>CGGGATTTGGCGATCGAATCTGATTGATCTAGATAATCCCTAGCGATCGCAAGTCCAAAGGTTGTCTACAATCAA<br>TATCCAAGCATCAAAAAGCGCCCCATTCGAGGCGCTTTTTGATTATTCAGACTGCTGTAATTCCGGCAATTAGGTT<br>ATTTGCCGACTACCTTGGTGATCTCGCCTTTCACGTAGTGGACAAATTCTTCCAACTGATCTGCGCGCGAGGCCA<br>AGCGATCTTCTTCTTGTCCAAGATAAGCCTGTCTAGCTTCAAGTATGACGGGCTGATACTGGGCCGGCAGGCGC<br>TCCATTGCCCAGTCGGCAGCGACATCCTTCGGCGCGATTTTGCCGGTTACTGCGCTGTACCAAATGCGGGACAA<br>CGTAAGCACTACATTTCGCTCATCGCCAGCCCAGTCGGGCGGCGAGTTCCATAGCGTTAAGGTTTCATTTAGCG<br>CCTCAAATAGATCCTGTTCAGGAACCGGATCAAAGAGTTCCTCCGCCGCTGGACCTACCAAGGCAACGCTATGT<br>TCTCTTGCTTTTGTCAGCAAGATAGCCAGATCAATGTCGATCGTGGCTGGCTCGAAGATACCAGCAAGAATGTCA<br>TTGCGCTGCCATTCTCCAAATTGCAGTTCGCGCTTAGCTGGATAACGCCACGGAATGATGTCGTCGTGCACAAC<br>AATGGTGACTTCTACAGCGCGGAGAATCTCGCTCTCTCCAGGGGAAGCCGAAGTTTCCAAAAGGTCGTTGATCA<br>AAGCTCGCCGCGTTGTTTCATCAAGCCTTACGGTCACCGTAACCAGCAAATCAATATCACTGTGTGGCTTCAGGC<br>CGCCATCCACTGCGGAGCCGTACAAATGTACGGCCAGCAACGTCGGTTCGAGATGGCGCTCGATGACGCCAAC<br>TACCTCTGATAGTTGAGTCGATACTTCGGCGATCACCGCTTCCCTCATAATGTTTAACTTTGTTTTAGGGCGACTG<br>CCCTGCTGCGTAACATCGTTGCTGCTCCATAACATCAAACATCGACCCACGGCGTAACGCGCTTGCTGCTTGGA<br>TGCCCGAGGCATAGACTGTACCCCAAAAAAACAGTCATAACAAGCCATGAAAACCGCCACTGCGCCGTTACCAC<br>CGCTGCGTTCGGTCAAGGTTCTGGACCAGTTGCGTGAGCGCATACGCTACTTGCATTACAGCTTACGAACCGAA<br>CAGGCTTATGTCCACTGGGTTCGTGCCTTCATCCGTTTCCACGGTGTGCGTCACCCGGCAACCTTGGGTAGCAG<br>CGAAGTCGAGGCATTTCTGTCCTGGCTGGCTAGGCTTAGGTCTTGGGATAGGCGATAGTAACGCCGTTGCCCCC<br>GTCAGCTTGATCGGCAGCTTCAAACTTCGCGACAAGTGGATGCTCGCGTAGGAACTGCTGCACGCCTTGCCGCA<br>GTTTGCCAGTGCCATGGCCATGGATGATCCAGATCGGTCCAATCGCTTGACGCAGCGCATCTTCCAGCACCACT<br>TCGGCTTCCGAAACACGACTGCCGCGTACATCGAAGGTATTGCGATCGGTGCGAATCGCCGGCGCATCTTTGG<br>GCGGGGGCGGCGGAGTTGTTGACTTAGGCGGGGGCGGGGGCAGTTCTACCTTTTCGCCCTGCAATGACTCCAC<br>ATCGCCGTAGCTGACCGTCAGCTTGAGGATGCCGCAACGCACCGTTAACTCCTGACGATCCGGCGCGATCGCA<br>AGGACTTCGCCGACCTTACCCAAGCGCGGAATCCGCAGGCGATCGCCCAACTGCGGCTGGAAACCAGGCGGT<br>GGGGCAACGACCGTCACGGCTGGTTGTGACAGCGACTTGAGGGTTTCACTGGCCTGCCGTGCTCGTTCTGCTT<br>TTGTCGCTTTCGGTCCCTGCTGCAGACGCCGCACCACCTTGGCAACCCGCTGTCGTGCCTGCTCAATTTCCGAC<br>TGGATCGCCACTTCTTGCTGCTGGCGCAAGCTTTGCTCTCGCTCCCGCAATTCGGCTGTCTTGGCTTGTAGTTC<br>GGCGTGTAGCTTTTCAGTCTGTTGCAACAACTGAGCAGCGGCGGCAGCCTTCTCTTCTTGATCGCGCCGTTGCG<br>CTTCCAGCCCTGCGATCACGGCATCTACATCGCGATCGCGGCCCCCTTCCAGTTGCGATCGCGCCTCAGCTAC<br>GACTGCTGGTGATAGTCCCAGTCGCTCGGCAATGATCAGCGCATTCGATCGCCCGGGGATGCCCCAGAGCAAG<br>CGATAGGTCGGAGAGAGGGTGCGATCGTCAAATTCTACCGAGGCATTTTCGAAGCGATCGTCCTGATATTTCAG<br>GGCCTT
L18q SeCS	GGCAGCAAACGCCGCAATCTCCAGATTCAAGCCACAGTACGGGGTGGCCATGGTCGCGATGGTGATTTTGAAGT<br>GTTTGCCACCTCCCTCGATCGCCTTGCGACAGCACTCGAAGGCCGCGATTCCCTGACGACAAATCCTGACTTCC<br>GCCAAGCCCAGCGCCAACTACCCCAACCCAATCAAGGTGTCCTCTACCTTGACTGGCAGGCCAGTGCCCTACC<br>GCTGACGCAACGCTGGCCTGTCTTGCAGCTGATTGCCCTGCCCTTGCAACCCCTGTTGGAGCACGTGCAGGCG<br>ATCGCGATCGCCAGTCCTACCCCTCCCGAAGACAGCACTGACCTGCAGCGACTCTCAATCGAACTGCTGTTGCG<br>GAGCTAGGCGATGGAACTCTATCTGATCCGCCATGGCATCGCCGCCGAACGGGGCACCTACGCGGATGATGAC<br>CGGCGCCCCCTGACAGCCACGGGAGAACGCCGCAGTCAACGAGTTGCCCAGCGACTGCTGTCTCTCGGTCTGC<br>ATTTCGATGTTCTGCAAACCAGTCCCCTAGTCCGCGCCCACCAAACCGCCGTCATCCTTCAACAAGAGGGGCTG<br>GCTTCCCAAATCGCGATCGCCCCAGAATTAGCGCCCGAAGGCAGTCTGACCGCTTGGCTCCGGCGCTTACCGC<br>CCGCCATCTCGGCTGATCAGCGCTGGGCGATCGTGGGCCATGAACCCGATCTAGGGGTCTGGGCGGAACAGCT<br>GGTCTGGGGATCTGCCCAGGACAAGCTCGTTCTCAAGAAAGCCGGTCTGATCGGTCTGCAATTCCCAGGCGATC

	GCCCTGCGATCGGAGCCGGATCCCTCTTCTGGCTAACCCCCCCGCGCCTTCTCCTTTAGGGGCACCTGATCAAA<br>TTGGCGAAAGACGCGCGGCAGACCCACCATCAGGTTTCGTGCCTGTTGCGACGAAGATGCACCAGCCAGACTG<br>ATAGGGTATTCGGAAGAAAGACTGCATGGGGAAGCCCAGGATTGCTATGACTGCCGTCAGCGAGTTTCGGCCTG<br>GCCTAGAAGGCGTGCCCGCCACACAGTCGAGCATTAGCTTTGTCGATGGCCAGCGCGGCGTCCTAGAGTATCG<br>CGGCATCAGCATCGAGCAACTGGCGCAACAGAGCAGTTTTCTGGAAACCGCCTACCTGTTGATTTGGGGCCATC<br>TACCAACTCAGCAGGAATTGACCGAGTTCGAGCACGAAATTCGCTACCACCGCCGCATCAAGTTCCGCATCCGG<br>GACATGATGAAATGCTTCCCCGATAGCGGCCATCCTATGGATGCCCTGCAGGCGAGCGCCGCAGCCCTCGGGT<br>TGTTCTATTCGCGGCGCGCCTTGGATGATCCCGAATACATTCGGGCGGCCGTTGTGCGTTTGCTAGCCAAAATT<br>CCGACGATGGTGGCTGCCTTCCAGCTGATCCGCAAGGGTAACGACCCAATTCAGCCCCGCGATGAACTGGACT<br>ACGCCGCCAACTTTCTCTACATGCTGACGGAGCGCGAGCCCGATCCAGTCGCAGCTCGGATTTTTGATATTTGC<br>CTCACCCTGCACGCCGAACATACGATCAACGCCTCGACCTTCTCGGCGATGGTCACAGCTTCGACCCTGACCGA<br>TCCCTACGCTGTCGTTGCTTCTGCCGTTGGCACCTTGGCTGGCCCCCTCCATGGCGGCGCCAATGAAGAAGTG<br>CTGGACATGCTGGAGGCGATCGGTTCCGTCGAGAATGTTGAGCCCTACCTCGACCACTGCATTGCCACCAAAAC<br>GCGCATTATGGGCTTTGGGCACCGTGTCTACAAAGTCAAGGATCCGCGGGCAGTCATTCTGCAAAATCTGGCCG<br>AGCAACTGTTCGATATCTTCGGCCATGATCCCTACTACGAAATCGCGGTCGCAGTCGAAAAGGCAGCAGCCGAG<br>CGACTCAGCCACAAGGGCATTTACCCCAACGTCGATTTCTACTCCGGCTTGGTCTATCGCAAGCTCGGTATTCCT<br>AGCGATCTATTCACACCGGTGTTTGCGATCGCGCGGGTTGCGGGCTGGCTCGCCCACTGGAAAGAGCAGCTGA<br>ACGAAAATCGGATCTTCCGCCCCACTCAGATCTACACGGGCAGCCACAACCTCGACTACACCCCGATCGCCGAT<br>CGGGATTTGGCGATCGAATCTGATTGATCTAGATAATCCCTAGCGATCGCAAGTCCAAAGGTTGTCTACAATCAA<br>TATCCAAGCATCAAAAAGCGCCCCATTCGAGGCGCTTTTTGATTATTCAGACTGCTGTAATTCCGGCAATTAGGTT<br>ATTTGCCGACTACCTTGGTGATCTCGCCTTTCACGTAGTGGACAAATTCTTCCAACTGATCTGCGCGCGAGGCCA<br>AGCGATCTTCTTCTTGTCCAAGATAAGCCTGTCTAGCTTCAAGTATGACGGGCTGATACTGGGCCGGCAGGCGC<br>TCCATTGCCCAGTCGGCAGCGACATCCTTCGGCGCGATTTTGCCGGTTACTGCGCTGTACCAAATGCGGGACAA<br>CGTAAGCACTACATTTCGCTCATCGCCAGCCCAGTCGGGCGGCGAGTTCCATAGCGTTAAGGTTTCATTTAGCG<br>CCTCAAATAGATCCTGTTCAGGAACCGGATCAAAGAGTTCCTCCGCCGCTGGACCTACCAAGGCAACGCTATGT<br>TCTCTTGCTTTTGTCAGCAAGATAGCCAGATCAATGTCGATCGTGGCTGGCTCGAAGATACCAGCAAGAATGTCA<br>TTGCGCTGCCATTCTCCAAATTGCAGTTCGCGCTTAGCTGGATAACGCCACGGAATGATGTCGTCGTGCACAAC<br>AATGGTGACTTCTACAGCGCGGAGAATCTCGCTCTCTCCAGGGGAAGCCGAAGTTTCCAAAAGGTCGTTGATCA<br>AAGCTCGCCGCGTTGTTTCATCAAGCCTTACGGTCACCGTAACCAGCAAATCAATATCACTGTGTGGCTTCAGGC<br>CGCCATCCACTGCGGAGCCGTACAAATGTACGGCCAGCAACGTCGGTTCGAGATGGCGCTCGATGACGCCAAC<br>TACCTCTGATAGTTGAGTCGATACTTCGGCGATCACCGCTTCCCTCATAATGTTTAACTTTGTTTTAGGGCGACTG<br>CCCTGCTGCGTAACATCGTTGCTGCTCCATAACATCAAACATCGACCCACGGCGTAACGCGCTTGCTGCTTGGA<br>TGCCCGAGGCATAGACTGTACCCCAAAAAAACAGTCATAACAAGCCATGAAAACCGCCACTGCGCCGTTACCAC<br>CGCTGCGTTCGGTCAAGGTTCTGGACCAGTTGCGTGAGCGCATACGCTACTTGCATTACAGCTTACGAACCGAA<br>CAGGCTTATGTCCACTGGGTTCGTGCCTTCATCCGTTTCCACGGTGTGCGTCACCCGGCAACCTTGGGTAGCAG<br>CGAAGTCGAGGCATTTCTGTCCTGGCTGGCTAGGCTTAGGTCTTGGGATAGGCGATAGTAACGCCGTTGCCCCC<br>GTCAGCTTGATCGGCAGCTTCAAACTTCGCGACAAGTGGATGCTCGCGTAGGAACTGCTGCACGCCTTGCCGCA<br>GTTTGCCAGTGCCATGGCCATGGATGATCCAGATCGGTCCAATCGCTTGACGCAGCGCATCTTCCAGCACCACT<br>TCGGCTTCCGAAACACGACTGCCGCGTACATCGAAGGTATTGCGATCGGTGCGAATCGCCGGCGCATCTTTGG<br>GCGGGGGCGGCGGAGTTGTTGACTTAGGCGGGGGCGGGGGCAGTTCTACCTTTTCGCCCTGCAATGACTCCAC<br>ATCGCCGTAGCTGACCGTCAGCTTGAGGATGCCGCAACGCACCGTTAACTCCTGACGATCCGGCGCGATCGCA<br>AGGACTTCGCCGACCTTACCCAAGCGCGGAATCCGCAGGCGATCGCCCAACTGCGGCTGGAAACCAGGCGGT<br>GGGGCAACGACCGTCACGGCTGGTTGTGACAGCGACTTGAGGGTTTCACTGGCCTGCCGTGCTCGTTCTGCTT<br>TTGTCGCTTTCGGTCCCTGCTGCAGACGCCGCACCACCTTGGCAACCCGCTGTCGTGCCTGCTCAATTTCCGAC<br>TGGATCGCCACTTCTTGCTGCTGGCGCAAGCTTTGCTCTCGCTCCCGCAATTCGGCTGTCTTGGCTTGTAGTTC<br>GGCGTGTAGCTTTTCAGTCTGTTGCAACAACTGAGCAGCGGCGGCAGCCTTCTCTTCTTGATCGCGCCGTTGCG<br>CTTCCAGCCCTGCGATCACGGCATCTACATCGCGATCGCGGCCCCCTTCCAGTTGCGATCGCGCCTCAGCTAC<br>GACTGCTGGTGATAGTCCCAGTCGCTCGGCAATGATCAGCGCATTCGATCGCCCGGGGATGCCCCAGAGCAAG<br>CGATAGGTCGGAGAGAGGGTGCGATCGTCAAATTCTACCGAGGCATTTTCGAAGCGATCGTCCTGATATTTCAG<br>GGCCTT
