# Supplementary material for: Emergence of fractal geometries in the evolution of a metabolic enzyme
Source: Nature. 2024 Apr 10;628(8009):894–900. doi: 10.1038/s41586-024-07287-2 (PMC11041685; doi:10.1038/s41586-024-07287-2)
Supplement: Supplementary file 2 — Reporting Summary [file 41586_2024_7287_MOESM2_ESM.pdf]

nature portfolio

Corresponding author(s): Georg Hochberg, Jan Schuller

Last updated by author(s): Feb 28, 2024

Reporting Summary

Nature Portfolio wishes to improve the reproducibility of the work that we publish. This form provides structure for consistency and transparency in reporting. For further information on Nature Portfolio policies, see our Editorial Policies and the Editorial Policy Checklist.

Statistics

For all statistical analyses, confirm that the following items are present in the figure legend, table legend, main text, or Methods section.

n/a	Confirmed	
☐	☒	The exact sample size (n) for each experimental group/condition, given as a discrete number and unit of measurement
☐	☒	A statement on whether measurements were taken from distinct samples or whether the same sample was measured repeatedly
☒	☐	The statistical test(s) used AND whether they are one- or two-sided<br>*Only common tests should be described solely by name; describe more complex techniques in the Methods section.*
☒	☐	A description of all covariates tested
☒	☐	A description of any assumptions or corrections, such as tests of normality and adjustment for multiple comparisons
☐	☒	A full description of the statistical parameters including central tendency (e.g. means) or other basic estimates (e.g. regression coefficient) AND variation (e.g. standard deviation) or associated estimates of uncertainty (e.g. confidence intervals)
☒	☐	For null hypothesis testing, the test statistic (e.g. F, t, r) with confidence intervals, effect sizes, degrees of freedom and P value noted<br>*Give P values as exact values whenever suitable.*
☒	☐	For Bayesian analysis, information on the choice of priors and Markov chain Monte Carlo settings
☒	☐	For hierarchical and complex designs, identification of the appropriate level for tests and full reporting of outcomes
☒	☐	Estimates of effect sizes (e.g. Cohen's d, Pearson's r), indicating how they were calculated

Our web collection on statistics for biologists contains articles on many of the points above.

Software and code

Policy information about availability of computer code

Data collection	Data collection was performed using the software provided by the respective instrument. Serial EM v3.8, AcquireMP v1.2.1, Tecan i-control 3.9.1. Amino acid sequences were collected from deposited sequences at the National Center for Biotechnology Information
Data analysis	ata analysis was performed using publicly available software as detailed in citations included in the manuscript and SI. MUSCLE v3.8.31 28, raxML v8.2.10, BOOSTER v0.1.0, PhyML 3.0, PAML v4.9, PAUP 4.0a, DiscoverMP v2.5.0, UniDec 4.0.2., GraphPad Prism 8.4.3, Excel Version 1808, Adobe Illustrator v24.0.2, MassHunter QQQ Quantative Analysis V10.0, cisTEM 1.0.0., XDS Version January 10, 2022; XSCALE Version January 10, 2022, PHASER 1.18.2-3874-000, WinCoot 0.9.6, PHENIX v1.19.2, cryoSPARC v3.2.0, v4.4.0 and v2.3, Gctf 1.06, Focus v1.0.0, RELION 3.1, ScÅtter IV, BioXTAS RAW 2.1.4, PyMOL 2.5.2, GROMACS 2022.2, AnAnaS v.0.6, Topaz v0.2.5, crYOLO v1.9.3, AlphaFold v2.1.2, UCSF Chimera v1.16, Namdinator v1.0

For manuscripts utilizing custom algorithms or software that are central to the research but not yet described in published literature, software must be made available to editors and reviewers. We strongly encourage code deposition in a community repository (e.g. GitHub). See the Nature Portfolio guidelines for submitting code & software for further information.

Data

Policy information about availability of data

All manuscripts must include a data availability statement. This statement should provide the following information, where applicable:

- Accession codes, unique identifiers, or web links for publicly available datasets
- A description of any restrictions on data availability
- For clinical datasets or third party data, please ensure that the statement adheres to our policy

Atomic structures reported in this paper are deposited to the Protein Data Bank under accession codes 8AN1, 8BP7, 8BEI, 8RJK and 8RJL. The cryo-EM data were deposited to the Electron Microscopy Data Bank under EMDB-15529, EMDB-16004, EMDB-19250 and EMDB-19251. All raw data for MP spectra, growth curves and kinetic traces as well as phylogenetic trees, alignments, and ancestral sequences are deposited on Edmond, the Open Research Data Repository of the Max Planck Society for public access and available under https://doi.org/10.17617/3.KNEQIR. NCBI reference sequence accession codes for the protein sequences that were experimentally investigated are found in the Supplementary Information (Supplementary Table 3). All NCBI reference sequence accession codes for protein sequences that were used for the evolutionary analysis are found in the multiple sequence alignment that is deposited in the Edmond repository.

Research involving human participants, their data, or biological material

Policy information about studies with human participants or human data. See also policy information about sex, gender (identity/presentation), and sexual orientation and race, ethnicity and racism.

Reporting on sex and gender	NA
Reporting on race, ethnicity, or other socially relevant groupings	NA
Population characteristics	NA
Recruitment	NA
Ethics oversight	NA

Note that full information on the approval of the study protocol must also be provided in the manuscript.

Field-specific reporting

Please select the one below that is the best fit for your research. If you are not sure, read the appropriate sections before making your selection.

☒ Life sciences ☐ Behavioural & social sciences ☐ Ecological, evolutionary & environmental sciences

For a reference copy of the document with all sections, see nature.com/documents/nr-reporting-summary-flat.pdf

Life sciences study design

All studies must disclose on these points even when the disclosure is negative.

Sample size	No sample size calculation was performed. For the inference of the phylogenetic tree aminoacid sequences of citrate synthase genes from cyanobacteria were collected from publicly deposited sequences. Sequences were selected in order to represent the diversity of the phylum and to follow the species phylogeny of cyanobacteria as reported in previous publications. For biochemical assays and growth curves 3 replicates were carried out to be able to calculate a standard deviation and keep the sample sizes experimentally manageable. For all Michaelis-Menten plots for the kinetic characterization of enzymes three independent experiments were performed on different days and set up with new substrate preparations. The measurements for the different substrate concentrations were done in three technical replicates for each of those three experiments. For growth curves and survival assays of S. elongatus strains as well as for metabolite extraction three biological replicates (i.e. independent cultures) in shaking flasks were used.
Data exclusions	For the inference of the phylogenetic tree of citrate synthases in cyanobacteria Prochlorothrix hollandica could not be recovered robustly as sister group to Synechococcus elongatus PCC 7942. Since this phylogenetic relationship is otherwise well established in the literature we excluded the sequence from the data set. We did use this sequence later to produce a constrained tree, with P. hollandica at the correct position, to verify the inference regarding the evolution of the fractal assembly (s. Extended Data Figure 8b).
Replication	Experiments were usually prepared in three biological replicates to ensure robustness of the conclusions. The exact number of replicates is always mentioned in the figure legend.
Randomization	NA
Blinding	NA

Reporting for specific materials, systems and methods

We require information from authors about some types of materials, experimental systems and methods used in many studies. Here, indicate whether each material, system or method listed is relevant to your study. If you are not sure if a list item applies to your research, read the appropriate section before selecting a response.

Materials & experimental systems

n/a	Involved in the study
☒	☐ Antibodies
☒	☐ Eukaryotic cell lines
☒	☐ Palaeontology and archaeology
☒	☐ Animals and other organisms
☒	☐ Clinical data
☒	☐ Dual use research of concern
☒	☐ Plants

Methods

n/a	Involved in the study
☒	☐ ChIP-seq
☒	☐ Flow cytometry
☒	☐ MRI-based neuroimaging

Plants

Seed stocks	*Report on the source of all seed stocks or other plant material used. If applicable, state the seed stock centre and catalogue number. If plant specimens were collected from the field, describe the collection location, date and sampling procedures.*
Novel plant genotypes	*Describe the methods by which all novel plant genotypes were produced. This includes those generated by transgenic approaches, gene editing, chemical/radiation-based mutagenesis and hybridization. For transgenic lines, describe the transformation method, the number of independent lines analyzed and the generation upon which experiments were performed. For gene-edited lines, describe the editor used, the endogenous sequence targeted for editing, the targeting guide RNA sequence (if applicable) and how the editor was applied.*
Authentication	*Describe any authentication procedures for each seed stock used or novel genotype generated. Describe any experiments used to assess the effect of a mutation and, where applicable, how potential secondary effects (e.g. second site T-DNA insertions, mosiacism, off-target gene editing) were examined.*
